# Radiomics as a personalized medicine tool in lung cancer: Separating the hope from the hype

**DOI:** 10.1016/j.lungcan.2020.05.028

**Published:** 2020-08

**Authors:** Isabella Fornacon-Wood, Corinne Faivre-Finn, James P.B. O’Connor, Gareth J. Price

**Affiliations:** a Division of Cancer Sciences, University of Manchester, Manchester, UK; b Department of Radiation Oncology, The Christie Hospital NHS Foundation Trust, Manchester, UK; c Department of Radiology, The Christie Hospital NHS Foundation Trust, Manchester, UK

**Keywords:** AUC, area under the curve, CI, concordance index, HR, hazard ratio, ROI, region of interest, RQS, radiomics quality score, TRIPOD, Transparent Reporting of a multivariable prediction model for Individual Prognosis Or Diagnosis, Radiomics, Imaging biomarkers, Lung cancer, Personalized medicine

## Abstract

Radiomics has become a popular image analysis method in the last few years. Its key hypothesis is that medical images harbor biological, prognostic and predictive information that is not revealed upon visual inspection. In contrast to previous work with *a priori* defined imaging biomarkers, radiomics instead calculates image features at scale and uses statistical methods to identify those most strongly associated to outcome. This builds on years of research into computer aided diagnosis and pattern recognition. While the potential of radiomics to aid personalized medicine is widely recognized, several technical limitations exist which hinder biomarker translation. Aspects of the radiomic workflow lack repeatability or reproducibility under particular circumstances, which is a key requirement for the translation of imaging biomarkers into clinical practice. One of the most commonly studied uses of radiomics is for personalized medicine applications in Non-Small Cell Lung Cancer (NSCLC). In this review, we summarize reported methodological limitations in CT based radiomic analyses together with suggested solutions. We then evaluate the current NSCLC radiomics literature to assess the risk associated with accepting the published conclusions with respect to these limitations. We review different complementary scoring systems and initiatives that can be used to critically appraise data from radiomics studies. Wider awareness should improve the quality of ongoing and future radiomics studies and advance their potential as clinically relevant biomarkers for personalized medicine in patients with NSCLC.

## Highlights

• Radiomics studies in NSCLC suffer from a number of limitations.
• No single radiomic signature has been translated into clinical use.
• Identification of limitations can help future studies to expedite biomarker translation.

## 1 Introduction

Lung cancer remains the leading cause of cancer-related mortality worldwide [1]. The 5 year survival for patients with non-small cell lung cancer (NSCLC), the most common form of the disease, is 10−20% [2,3]. Despite advances in treatment options in recent years, survival rates have changed little [3,4].

Given the patient variability and tumor heterogeneity of this cancer, personalizing treatment is key to improving survival beyond the current poor prognosis [5]. One requirement for successful delivery of personalized medicine is the identification and validation of biomarkers that can predict which patients will benefit from a given therapy. There is an unmet need for such biomarkers in lung cancer [6].

Medical imaging plays a key role in the diagnosis and treatment of lung cancer, making the use of image-based biomarkers to guide clinical decision-making attractive. Over the last several decades, a number of biomarkers derived from CT, PET and MRI that measure tumor size, shape and texture, or quantify aspects of the tumor microenvironment have been used in lung cancer studies for diagnosis, prediction, prognostication and response monitoring [[6], [7], [8]].

**Fig. 1:**
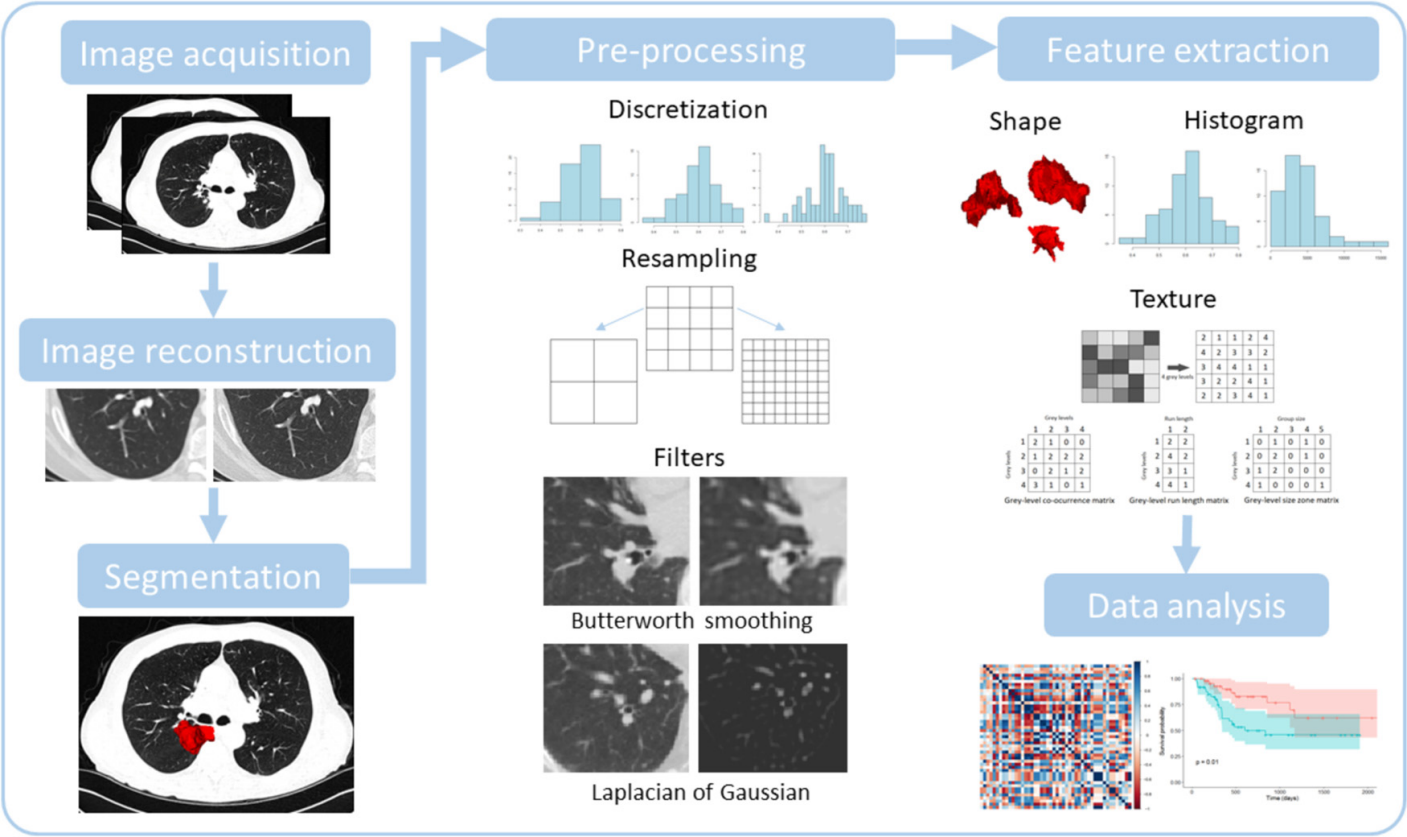
Visualization of the steps in the radiomics workflow. First, images are acquired and reconstructed. The region of interest is then segmented, from which features will be extracted. Next, pre-processing steps are performed to modify the images before feature extraction. Shape, first order (or histogram) and texture features are then extracted from the region of interest. Finally, data analysis steps attempt to find correlations between features and the specified outcome.

There is currently substantial interest in using computer algorithms to extend this approach to extract tens to thousands of image ‘features’ in an analysis pipeline strategy termed ‘radiomics’. Such methods test the hypothesis that medical images harbor data that will provide biomarkers for personalized medicine, but that the optimum biomarkers are not readily determined *a priori* [9]. Imaging biomarker studies postulate that medical images contain biological, prognostic and predictive information that is not apparent when clinicians view scans [10]. In radiomics, this information is extracted from digital images using computer algorithms to form ‘radiomic signatures’, a type of quantitative imaging biomarker formed by combining the radiomics features that have the strongest association to the measured outcome. The radiomics workflow consists of a series of steps [11]. summarized in Fig. 1. Proponents of radiomics hypothesize that these data-driven approaches will select the most statistically significant signature that relates to an outcome measure of interest. This approach is extremely popular, but to date the resultant imaging biomarkers have not been validated as useful tools for personalized medicine [12].

CT is the most commonly used modality worldwide for diagnosis, treatment planning, and follow-up in all stages of lung cancer, meaning that informative imaging biomarkers discovered from these data could be translated rapidly into clinical practice. In this review, we summarize the literature supporting use of CT radiomic biomarkers to guide decision-making in patients with NSCLC.

We appraise the published reports of CT radiomics biomarkers as predictive, prognostic or biologically informative tools and review literature highlighting methodological limitations. Our aims are to evaluate how robust the conclusions of these studies are and to assess how well the current standardization and reporting tools inform readers of the potential limitations when interpreting their results.

## 2 The potential of radiomics for personalized decision-making in NSCLC

A review of the literature found 43 CT image based studies that evaluated the prognostic or predictive role of radiomic signatures in patients with NSCLC (Table 1). Three of these studies, together with a further 21 we separately identified, evaluated the role of radiomic signatures in appraising aspects of tumor biology including genomic or pathologic biomarkers, signalling pathways, and disease classification in NSCLC (Table 2).

**Table 1:** Radiomics studies in NSCLC, categorized into sections based on their investigated endpoint. The Data column specifies the total number of patients involved in the study, in brackets split by training and validation cohorts if applicable and specifying other cancer types of cohorts if applicable. Note: Studies marked with * are validation studies and their RQS score components refer to methodology based on the previous published data. This table has been simplified to clarify presentation – more details for each study are available in Supplementary Table 2.

Reference	NSCLC stage	Data (training + validation)	Radiomic features in final model	Result
**Overall survival**				
Aerts et al. 2014 [13]	1−3b	647 pCT (422 + 225)	Shape, first order and texture	CI = 0.65
Van Timmeren et al. 2017 [14]*	1−4	252 pCT and CBCT (102 + 56 + 94)	Shape, first order and texture	CI = 0.69, 0.61, 0.59 (pCT) CI = 0.66,0.63,0.59 (CBCT)
Grossman et al. 2017 [15]*	1−3	351 diagnostic CT (262 + 89)	Shape, first order and texture	CI = 0.60
Grossman et al. 2017 [15]	1−3	351 diagnostic CT (262 + 89)	Not specified	CI = 0.61
Yu et al. 2017 [16]	1	442 diagnostic CT (147 + 295)	First order and texture	CI = 0.64
Chaddad et al. 2017 [17]	1−3b	315 pCT	Shape and texture	Average AUC = 0.70−0.76
Fave et al. 2017 [18]	3	107 4DCT end of exhale, pCT and CBCT	Shape and texture	CI = 0.672
Li et al. 2017 [19]	1−2a	59 follow up CT	Texture	AUC = 0.81
Li et al. 2017 [20]	1−2a	92 4DCT Average-CT or 50 % phase-CT	Shape and first order	AUC = 0.728
Tang et al. 2018 [21]	1−3	290 staging CT (114 + 176)	Shape, first order and texture	CI = 0.72
Bianconi et al. 2018 [22]	1−3	203 pCT	Shape and texture	HR = 1.06−1.48
De Jong et al. 2018 [23]*	4	195 diagnostic CT	Shape, first order and texture	CI = 0.576
Lee et al. 2018 [24]	1−3	339 CT	Shape, first order and texture	CI = 0.772
He et al. 2018 [25]	1−3	186 CT (298 after oversampling (223 + 75))	Not specified	AUC = 0.9296
Starkov et al. 2018 [26]	1	116 pCT	Texture	High risk vs low risk median p-values = 0.04–0.07
Yang et al. 2018 [27]	1−4	371 CT (239 + 132)	First order and texture	CI = 0.702
Wang et al. 2019 [28]	3	70 pre-treatment and 97 post-treatment CT from 118 patients	Texture	CI = 0.743
Shi et al. 2019 [29]	3	11 CBCT from 23 patients	First order	HR = 0.21
Van Timmeren et al. 2019 [30]	1−4	337 pCT and 2154 CBCTs from 337 patients (141 + 94 + 61 + 41)	First order and texture	CI = 0.59, 0.54, 0.57
Huang et al. 2019 [31]	1−4	371 CT (254 + 63 + 54)	Shape, first order and texture	CI = 0.621, 0.649
Franceschini et al. 2019 [32]	1−2	102 4DCT start of inspiration (70 + 32)	Shape and texture	AUC = 0.85
**Local or metastatic recurrence**				
Coroller et al. 2015 [33]	2−3	182 pCT (98 + 84)	First order and texture	CI = 0.6
Mattonen et al. 2016 [34]	1	45 follow-up CT	First order and texture	AUC = 0.85
Huynh et al. 2016 [35]	1−2	113 CT	First order and texture	Median CI = 0.67
Huynh et al. 2017 [36]	1−2a	112 CT and AIP CT	Shape, first order and texture	AIP radiomics CI = 0.667 FB radiomics CI = 0.601
Fave et al. 2017 [18]	3	107 4DCT end of exhale, pCT and CBCT	Shape and texture	CI = 0.632, 0.558 (DM, LRR)
Li et al. 2017 [19]	1−2a	59 follow up CT	Texture	AUC = 0.80, 0.80 (RFS, LR-RFS)
Li et al. 2017 [20]	1−2a	92 4DCT Average-CT or 50 % phase-CT	Shape	AUC = 0.747, 0.690 (RFS, LL-RFS)
Dou et al. 2018 [37]	2−3	200 pCT (100 + 100)	Texture	CI = 0.65
Ferreira Junior et al. 2018 [38]	1−4	68 CT (52 + 16)	Shape and texture	AUC = 0.75, 0.71 (lymph node metastasis, DM)
Yang et al. 2018 [39]	1−3	159CT (106 + 53)	Shape, first order and texture	AUC = 0.856
Zhong et al. 2018 [40]	1−2	492 CT	First order and texture	AUC = 0.972
Lafata et al. 2019 [41]	1	70 CT	Texture	Maximum AUC = 0.72, 0.83, 0.60 (recurrence, LR, non-LR)
Akinci D’Antonoli et al. 2019 [42]	1−2b	124 CT	Shape, first order and texture	AUC 0.731, 0.750 (LR, DM)
He et al. 2019 [43]	Not specified	717CT (423 + 294)	First order and texture	CI = 0.734
Xu et al. 2019 [44]	3−4	132 CT (106 + 26)	Texture	AUC = 0.642
Franceschini et al. 2019 [32]	1−2	102 4DCT start of inspiration (70 + 32)	Shape, first order and texture	AUC = 0.73
Ferreira-Junior et al. 2019 [45]	1−4	85 CT	Shape, first order and texture	AUC = 0.92, 0.84 (DM, nodal metastasis)
Cong et al. 2019 [46]	1a	649 venous phase CT (455 + 194)	Shape, first order and texture	AUC = 0.851
**Treatment response, disease-free or progression-free survival**				
Coroller et al. 2016 [47]	2−3	127 pCT	Shape, first order and texture	Median AUC = 0.65, 0.61 (GRD, pCR)
Huang et al. 2016 [48]	1−2	282 CT (141 + 141)	First order and texture	HR = 2.09
Song et al. 2016 [49]	1−4	152 CT (80 + 72)	Texture	HR = 2.35, 2.75
Coroller et al. 2017 [50]	2−3	85 pCT	Shape, first order and texture	Median AUC = 0.68, = 0.71 (pCR, GRD)
Tunali et al. 2019 [51]	3b-4	228 CT	Texture	AUC = 0.804
Franceschini et al. 2019 [32]	1−2	102 4DCT start of inspiration (70 + 32)	Texture	AUC = 0.88
**Lung toxicity**				
Moran et al. 2017 [52]	1	14 diagnostic CT	First order and texture	AUC = 0.689−0.750
Krafft et al. 2018 [53]	Not specified	192 50 % 4DCT phase	First order and texture	Average AUC = 0.68
**Staging**				
Yuan et al. 2018 [54]	1	327 CT	First order and texture	AUC = 0.938
Yang et al. 2019 [55]	1−3	256 CT	First order and texture	AUC = 0.93

Abbreviations: AUC, area under the curve; CBCT, cone-beam CT; CI, concordance index; DFS, disease free survival; DM, distant metastasis; GRD, gross residual disease; H&N, head and neck; HR, hazard ratio; LR, local relapse; LRR, local regional recurrence; LR-RFS, loco-regional recurrence-free survival; OS, overall survival; pCR, pathological complete response; pCT, radiotherapy planning CT scan; PFS, progression free survival; RFS, recurrence free survival.

**Table 2:** Radiomics studies in NSCLC with an aspect of biology as the endpoint. The column labeled ‘Data’ specifies the total number of patients involved in the study, in brackets split by training and validation cohorts if applicable and specifying other cancer types of cohorts if applicable. This table has been simplified to clarify presentation – more details for each study are available in Supplementary Table 3.

Reference	Stage	Endpoint	Data (training + validation)	Radiomic features in final model	Result
**Genomics**					
Aerts et al. 2016 [56]	Early stage	EGFR	47 diagnostic CT and follow-up	Shape and texture	AUC = 0.74−0.91
Rios Velazquez et al. 2017 [57]	1−4	EGFR, KRAS	705 diagnostic CT (353 + 352)	Shape, first order and texture	AUC = 0.69−0.80
Mei et al. 2018 [58]	Not specified	EGFR	296 CT	Texture	AUC = 0.664
Digumarthy et al. 2019 [59]	Not specified	EGFR	93 CT	First order	AUC = 0.713
Jia et al. 2019 [60]	1−4	EGFR	504 CT (345 + 158)	Shape, first order and texture	AUC = 0.802
Li et al. 2019 [61]	1−4	EGFR subtypes (19Del and L858R)	312 CT (236 + 76)	Shape, first order and texture	AUC = 0.775−0.793
Tu et al. 2019 [62]	1−4	EGFR	404 CT (243 + 161)	First order and texture	AUC = 0.775
Yang et al. 2019 [63]	Not specified	EGFR	467 CT (306 + 161)	Shape, first order and texture	AUC = 0.789
Wang et al. 2019 [64]	1−2	EGFR, TP53	61 CT (41 + 20)	First order and texture	AUC = 0.604, 0.586
Wang et al. 2019 [64]	1−2	Tumor mutation burden	61 CT (41 + 20)	Texture	AUC = 0.606
**Signaling pathways**					
Grossman et al. 2017 [15]	1−3	Various	351 CT (262 + 89)	Shape, first order and texture	AUC = 0.62−0.72
Bak et al. 2018 [65]	1−4	Various	57 CT	First order and texture	OR = 0.08−23.94
**Histopathology**					
Patil et al. 2016 [66]	Not specified	ADC, LCC, SCC, NOS	317 pCT	Shape, first order and texture	88 % accuracy
Wu et al. 2016 [67]	1−4	ADC, SCC	350 pCT (198 + 152)	First order and texture	AUC = 0.72
Ferreira Junior et al. 2018 [38]	1−4	ADC, SCC	68 CT (52 + 16)	Not specified	AUC = 0.81
Zhu et al. 2018 [68]	Not specified	ADC, SCC	129 CT (81 + 48)	First order and texture	AUC = 0.893
Digumarthy et al. 2019 [59]	Not specified	ADC, SCC	93 CT	First order	AUC = 0.744
E et al. 2019 [69]	Not specified	ADC, SCC, SCLC	229 CT	Shape, first order and texture	AUC = 0.657−0.875
Ferreira-Junior et al. 2019 [45]	1−4	ADC, SCC	85 CT	Shape, first order, texture	AUC = 0.88
Liu et al. 2019 [70]	Not specified	ADC, LCC, SCC, NOS	349 CT (278 + 71)	Not specified	AUC = 0.86
Zhou et al. 2018 [71]	1−4	Ki-67	110 CT	Shape and texture	AUC = 0.61−0.77
Gu et al. 2019 [72]	Not specified	Ki-67	245 CT	First order and texture	AUC = 0.776
Song et al. 2017 [73]	1−3	Micropapillary pattern	339 CT	First order	AUC = 0.751
Chen et al. 2018 [74]	Not specified	Degree of differentiation	487 CT (303 + 184)	First order and texture	AUC = 0.782
She et al. 2018 [75]	Not specified	Invasive vs non-invasive adenocarcinoma	402 CT (207 + 195)	Shape, first order and texture	AUC = 0.89
Yang et al. 2019 [76]	Not specified	Invasive vs non-invasive adenocarcinoma	192 CT (116 + 76)	First order and texture	AUC = 0.77

Abbreviations: ADC, adenocarcinoma; AUC, area under the curve; CI, concordance index; EGFR, epidermal growth factor receptor; KRAS, Kirsten rat sarcoma viral oncogene homolog; LCC, large cell carcinoma; NOS, not otherwise specified; OR, odds ratio; SCC, squamous cell carcinoma.

**Fig. 2:**
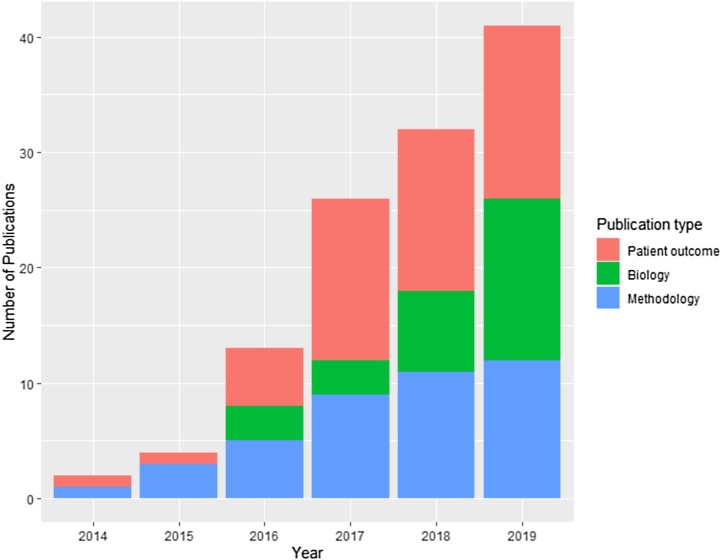
Frequency of CT NSCLC radiomics studies published from 2014 to 2019. Publications are categorized as those investigating radiomics methodological concerns, those evaluating radiomic signatures as prognostic or predictive biomarkers of patient outcome, and those evaluating radiomic signatures as biomarkers of tumor biology.

In addition, 42 studies reported on radiomics methodological limitations, potential problems, and possible solutions in CT based studies using data from NSCLC patients or imaging phantoms. The frequency of publications, for all types of NSCLC radiomics study, has markedly increased over the last six years (Fig. 2). Our search strategies are described in detail in Supplementary Materials.

The initial studies labelled as ‘radiomics’ were published in 2014 and 2015. Aerts and colleagues showed that a radiomic signature based on shape and texture metrics was associated with overall survival, validating the signature in patients with NSCLC and patients with head and neck cancers [13]. The study also found positive associations between the radiomic signature and gene expression. Coroller and colleagues showed that a different set of texture metrics were associated with the subsequent development of distant metastases [33]. The hypothesized mechanism was that tumor heterogeneity, identified by the radiomics analyses, drives worse outcomes. Both studies were performed using radiotherapy planning CT data.

Over the next four years (2015–2019), 41 CT studies were published that linked radiomics to lung cancer patient outcome. In general, studies sought to evaluate whether or not radiomic signatures could outperform existing methods for patient risk stratification. 20 studies related radiomics to overall survival [[13], [14], [15], [16], [17], [18], [19], [20], [21], [22], [23], [24], [25], [26], [27], [28], [29], [30], [31], [32]], 18 to the likelihood of local or metastatic recurrence [[18], [19], [20],[32], [33], [34], [35], [36], [37], [38], [39], [40], [41], [42], [43], [44], [45], [46]], 6 to response, disease-free or progression-free survival [32,[47], [48], [49], [50], [51]], and 2 to staging [54,55]. Two further studies focused on the association of radiomics signatures to lung toxicity [52,53], Four studies investigated multiple endpoints.

The majority of studies derived radiomics signatures in radiotherapy planning or diagnostic images acquired prior to therapy. Nearly all studies evaluated patients undergoing treatment with cytotoxic chemo-radiotherapy. More recently, a number of studies have evaluated the potential of radiomics to improve patient stratification for targeted therapies and immunotherapy agents [21,51,56]. For example, Tang and colleagues linked radiomic features to a tumor immune phenotype in patients with stage I-III NSCLC, finding patients with heterogeneous tumors, which correlated with low PD-L1 and high CD3 cell count, had better prognosis [21].

There are 24 CT studies evaluating how radiomic signatures of NSCLC relate to genomics [[56], [57], [58], [59], [60], [61], [62], [63], [64]], signalling pathways [15,65] and histopathology [38,45,59,[66], [67], [68], [69], [70], [71], [72], [73], [74], [75], [76]]. For example, Rios Velazquez and colleagues found distinct imaging phenotypes for EGFR and KRAS mutations from CT images of patients with NSCLC [57]. Some of the studies that relate radiomics to patient outcome also relate their radiomic signature to genomics [13] or biological markers [28].

Collectively, these 64 studies present a positive view of the potential for radiomics signatures to deliver personalized medicine. However, two important limitations are readily apparent. Firstly, while nearly all studies report at least one positive association between CT radiomic signature and either outcome (OS, PFS, recurrence or toxicity) or tumor biology (genomic or pathology biomarkers and signalling pathways), the particular radiomic signature derived varies substantially between studies. Consequently, few study signatures are directly comparable with one another, and so the literature does not identify specific candidate radiomic signatures for further large multicenter evaluation.

Secondly, it has become clear that studies can suffer from significant technical limitations. Studies of these limitations have also increased over the last five years, although at a slower pace than the patient outcome studies (Fig. 2).

## 3 Reported methodological limitations of CT based radiomics studies

All biomarkers, including radiomic signatures, must undergo technical and biological validation to become robust tools used to guide clinical decision-making. These validation steps take a biomarker from discovery to research assay where the biomarker can be used with confidence to determine an outcome in a research setting (termed ‘crossing translational gap 1′). The regulatory approval process (through e.g. the FDA or EMA) then takes the biomarker from research assay to clinically approved assay for use in decision-making in patients (termed ‘crossing translational gap 2′) [12].

To date, very few radiomics signatures have crossed either of these translational gaps. The first radiology product with radiomics capabilities to receive such approvals was QuantX for detection of breast abnormalities based on MRI, receiving FDA approval in 2017 [77]. Soon afterwards, Feedback Medical received CE approval for TexRAD Lung, a quantitative image texture analysis technology [78].

In this section, we evaluate the methodological limitations preventing CT based radiomics signatures from crossing these translational gaps. We review the potential problems and proffered solutions identified in 42 studies of imaging phantoms or patients with NSCLC (summarized in Table 3 and expanded in Supplementary Table 1).

**Table 3:** Potential problems at each step of the radiomics workflow along with possible solutions offered by the literature. Each workflow step with potential problems and solutions identified by the literature is labelled with a letter A-H to reference in-text. Note: Modelling does not have a letter associated with since there is no consensus on the best statistical modelling strategies.

Problem area		Potential problems	Potential solutions
Image acquisition	A	Different scanners and acquisition protocols affect feature reproducibility [[79], [80], [81], [82], [83], [84], [85], [86], [87], [88], [89], [90], [91]]	Image phantoms on different scanners to provide baseline [79], establish credibility of scanners and protocols [84], catalogue reproducible features [86,90], model a correction algorithm [89], harmonize data [91].
	B	Patient motion affects feature reproducibility [80,92,93]	Set motion tolerances, reduce ROI boundaries [80], use single phase from 4D images [92], find robust features using 4DCT data [93].
Image acquisition and reconstruction	C	Image resolution parameters (voxel size, slice thickness) affect feature values [79,88,[94], [95], [96], [97], [98]] model performance [99].	Control resolution [79] parameters in prospective studies, resample to common resolution and voxel depth [[94], [95], [96],^98^], apply smoothing image filters [95], apply deep learning methods [100].
Image reconstruction	D	Image reconstruction algorithm and reconstruction parameters (kernel) affects features [97,101,102]	Pre-processing image correction [101] and harmonization of acquisition techniques [97,102].
Segmentation	E	Delineation variability [90,[103], [104], [105], [106], [107]] affects features and is time consuming [106,107]. Results from one disease site are not necessarily transferrable to another [108].	Expert ROI definition [103], multiple observers [103,104,108], identification of stable features with respect to delineation [90,104,105], automated segmentation [106,107], image filtering [108]
Pre-processing	F	Number of grey levels used to discretize histogram and texture features affects feature values [96,98,109], as does bin width [94].	Texture features can be normalized to reduce dependency on the number of grey levels [98], number of grey levels used for discretization should be recorded with feature formula. 128 grey levels may be optimal for texture features, along with thresholding [109]
Feature extraction		No studies found in the literature search.	
Feature correlation	G	Strong correlations between tumor volume and radiomic features exist [98,[110], [111], [112]]	Normalization of features to volume [98], bit depth resampling [110], feature redesign [110], more robust statistics to check added value of radiomics signatures [111].
Test re-test	H	Radiomic features may not be repeatable over multiple measurements [[113], [114], [115]], repeatable features are not generalizable to other disease sites [116].	Test-retest data acquisition [113,116], use of multiple 4D phases [113,115], use of simulated retest by image perturbation [114].
Modelling clinical outcome		Different modelling strategies affect model performance [[117], [118], [119], [120]]	Sample sizes above 50 give better predictive performance [118], as does normalizing features [117]. No consensus on best modelling strategies to use.

### 3.1 Image acquisition

Many radiomics studies are retrospective evaluations of CT images, often with data acquired at multiple different institutions and on different CT scanner vendor platforms. Consequently, nearly all studies contend with variations in image acquisition and reconstruction protocols.

Studies assessing the impact of different CT scanners and protocols on radiomic features have shown some features have poor reproducibility [79,80,[84], [85], [86], [87]]. Performing phantom studies on different scanners as a quality assurance step may ensure a level of feature consistency [84]. Indeed, one study showed that using a controlled protocol across different CT scanners reduced feature variability by over 50 % compared to using local protocols [79]. Other studies used post-extraction deep learning [100] or correction factors [89] to reduce feature variability.

Restricting study data to one scanner make and model along with one set of acquisition parameters, to reduce variability in image capture acquisition, is seldom feasible for a multicentre research study. Therefore, many of these issues still remain when setting up a well powered prospective clinical trial with radiomic signatures as exploratory endpoints.

### 3.2 Image reconstruction

Retrospective data analyses are constrained by image reconstruction parameters determined by clinical department protocols, chosen to optimize image anatomical quality. While variations in image reconstruction, slice thickness and in plane pixel dimensions may have negligible effect for clinical interpretation, they can induce variability in radiomic feature values, since many features correlate to these parameters [79,[94], [95], [96], [97], [98]].

Resampling the image to an equal voxel size has reduced feature dependency on acquisition in some studies [94,96] but not others [79,95]. Smoothing filters have also been suggested as a method for reducing voxel size dependency [95], as has limiting inclusion criteria to particular resolution ranges. For example, Lu et al. found that features calculated from images with 1.25 mm and 2.5 mm thick slices were comparable to each other but that both differed from those calculated on 5 mm slice thickness images [97].

Reconstruction techniques also influence feature values with studies demonstrating differences between features calculated on images reconstructed with soft or sharp kernels [97,102]. Potential solutions include the application of correction factors based on the image noise power spectrum [101]. Solutions that balance feature robustness with the need to make image inclusion criteria as permissive as possible are vital given the small cohorts size issues that blight many studies.

### 3.3 Segmentation

The ROI definition for feature extraction is known to be a particularly sensitive step in the radiomics pipeline [[103], [104], [105], [106], [107]]. Radiomics studies are popular in radiotherapy given the ready availability of pre-defined ROIs on treatment planning scans, typically using the clinically defined Gross Tumor Volume (GTV). The subjectivity of GTV definition can depend on the operator, as expert delineations may generate features with better predictive power than those from a non-specialist [103].

Frequently suggested solutions include the inclusion of multiple observers or the use of semi-automated delineation tools [106,107]. However, few studies have adopted these solutions, most likely due to the difficulty of getting clinically qualified staff to delineate ROIs. In studies not using radiotherapy planning CT scans, the ROIs must be drawn specifically for the purpose of the radiomics analysis and will suffer from all of the same issues discussed above.

### 3.4 Pre-processing

The preparation of images for feature extraction has a marked effect on feature value. Reducing the number of image grey-levels (voxel depth re-binning) is a commonly used method to supress image noise. However, studies have shown that radiomic features are not comparable when computed with a differing intensity bin sizes [94,96,98]. This has led to the proposed use of standardized bin resolution [98].

### 3.5 Feature extraction

Radiomics features span a range of calculation classes. Shape features contain information about the ROI morphology (such as volume and measures of sphericity). First-order image intensity features assess properties of the intensity histogram of voxels within the ROI (e.g. the mean intensity and other statistical moments of the histogram). Texture features summarize different measures of the way in which voxel intensities change across the ROI (e.g. voxel variation coarseness and homogeneity). These features may be calculated on the original image or derived after various filters have been applied that modify particular aspects of it, for example to enhance the edges where image intensity changes [11].

Many different software platforms exist for performing the feature extraction step, including free open-source software, commercial software, and software developed in-house by individual institutions. The Image Biomarker Standardization Initiative (IBSI) is an international collaboration between research groups with the aim of standardizing image biomarker extraction [121]. To date only one study has investigated whether feature extraction software influences radiomic features from CT scans of patients with NSCLC [122], which shows, consistent with data from other cancer types [123,124], that this can have substantial impact on feature values.

### 3.6 Feature correlation

Since many tens to thousands of features are calculated from images in radiomics, it is unsurprising that many features often correlate with one another. However, the fact that features often correlate strongly with tumor volume and clinical factors [98,110,111] is not well appreciated. While it has been suggested that radiomic feature calculations formulae should be modified to be account for tumor volume [98], it is crucial that studies also include transparent and robust feature reduction steps to account for other clinical prognostic and predictive factors. Robust feature reduction is also crucial in limiting the risk of model overfitting.

### 3.7 Test-retest

As highlighted by several studies, [113,116] and by consensus statements on imaging biomarkers [12], radiomics studies usually lack an assessment of the signatures’ single centre repeatability or multicentre reproducibility. The use of test-retest datasets in which multiple images of the same subjects or phantom have been acquired in quick succession have been proposed as a means to assess repeatability [113,116]. Alternative options include the use of multiple 4D image phases [113] and the simulation of retest data by image perturbation [114] where test-retest data are not available. Few radiomic studies incorporate any of these approaches.

### 3.8 Modelling clinical outcome

Typically, studies derive between tens to a few thousand image features in development datasets [125]. Dimensionality reduction to remove highly correlated and unstable radiomic features is often employed before finding the most informative features for a specific outcome, such as overall survival, treatment-related toxicities or cancer recurrence in a test dataset. Many different statistical options exist for deriving a model based on radiomic features. The choice of model and statistical methods can influence results [[118], [119], [120]].

Random forests have been found by some authors to give higher performance compared to other methods for classification tasks using radiomics features [118,120], with Naïve Bayes and Support Vector Machines also reported to perform well [118]. For radiomic feature based time-to-event analyses, one study found cox regression with gradient boost performed better than traditional cox regression (0.614 versus 0.660 concordance index) [119]. In terms of feature selection, there is no consensus on the best method to use. Optimal performance of feature selection techniques depend on the outcome of interest [118]. A contemporary non-radiomics study of classifier performance in radiotherapy datasets found that random forest and elastic net logistic regression performed best, but that classification accuracy depended on the specific dataset [126]. To summarize, there is limited consensus as to the best machine learning methods to employ for radiomics studies, and that the optimum choice may depend on the specific dataset used in the study.

Regardless of feature selection and modelling methodology, the resulting model (often termed a ‘radiomic signature’) should be robustly validated in line with the TRIPOD guidelines to ascertain if it is reproducible across different clinical datasets. This tests if the observed signature relates to the desired outcome in a different patient group, and aims to reduce the risk of overfitting in the training cohort [125].

Lastly, whatever approach is taken it is vital that investigators test whether incorporating radiomic features into a clinical model adds any benefit to well-known clinical prognostic factors such as tumor stage and performance status. Radiomic features will only have clinical utility if they provide more predictive information than is currently available in the clinic.

## 4 Assessing the quality of radiomics studies in NSCLC

We evaluated the quality of the 43 radiomics studies we identified that report a relationship between a CT defined radiomic signature and clinical outcome in patients with NSCLC (Supplementary Table 2) using both established assessment tools and the results of our review of methodological limitations reported above. We then applied the same tools to the 24 studies that evaluated the relationship between CT radiomic signatures and genomic, protein expression, and pathology biomarkers in patients with NSCLC (Supplementary Table 3). Some studies investigated multiple endpoints, so in total we evaluated 75 outcomes. The four tools we use to interpret the technical validation of these studies are:

1 The strength of the validation in each study, assessed by the Transparent Reporting of a multivariable prediction model for Individual Prognosis Or Diagnosis (TRIPOD) guidelines [127]. TRIPOD provides an ordinal score (1−4, with 4 being the most robust). These guidelines are not specific to radiomics studies, but provide insight into the level of validation in a study (details in Supplementary Table 4).
2 The Radiomics Quality Score (RQS) developed by Lambin and colleagues [128]. RQS provides a checklist to evaluate aspects of study design, by assessing various technical and statistical aspects of the radiomics pipeline. It consists of 16 components, each of which award or penalize points, to provide the RQS. The total number of points available range from -8 to 36 (the more points the better) and are often presented as a percentage (Supplementary Table 5).
3 Qualitative assessment of radiomics methodological limitations resulting from our literature review and labelled as A–H and listed in Table 3.
4 The reported evidence for added value of the radiomics signature to a clinical model of outcome tested in the study (for the patient outcome studies only). This provides an assessment of clinical utility.

## 5 Interpreting the quality of radiomics studies in NSCLC

Studies linking CT radiomics signatures to clinical outcome and tumor biology were found to have a high incidence of methodological limitations (summarized in Table 4). Overall, half of studies had a TRIPOD type of either 1a or 1b (meaning the results were not validated or validated within the same dataset). Only 13/75 studies had TRIPOD type of 3 or 4 (meaning the results were validated in an external dataset). The median RQS was 6 (range of -8 to 36). Details on RQS and TRIPOD are found in Supplementary Material. We found that 70 % of studies (52 of 75) had six or more methodological limitations, and no study had less than three methodological limitations. Finally, over half of studies relating radiomics to patient outcome did test the added benefit of the radiomic signature to a clinical model.

**Table 4:** Summary of the 4 assessment criteria - TRIPOD score, RQS, number of methodological limitations and testing the added value of radiomics to a clinical model. The added value of radiomics to a clinical model was only tested for the patient outcome studies (N = 50).

	N = 75
TRIPOD type (n (%))	
1a – no validation	10 (13)
1b – internal validation	27 (36)
2a – dataset randomly split for validation	18 (24)
2b – dataset non-randomly split for validation	7 (9)
3 – external validation	10 (13)
4 – validation only	3 (4)
RQS (median, [IQR])	6 [2−12.25]
Number of methodological limitations (n (%))	
0−2	0 (0)
3	4 (5)
4	4 (5)
5	15 (20)
6	21 (28)
7	23 (31)
8	8 (11)
	N = 50
Added value of radiomics to clinical model tested? (n (%))	
Yes	32 (64)
No	18 (36)

**Fig. 3:**
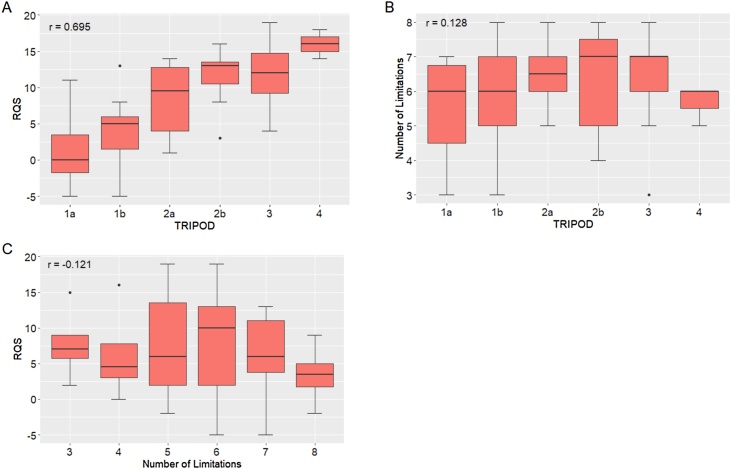
The assessment of the literature plotted against each other as boxplots. (A) RQS versus TRIPOD, (B) RQS versus the number of methodological limitations found in this review and (C) TRIPOD versus the number of methodological limitations found in this review.

Our analysis suggests that the four assessment tools provide useful and complimentary critiques. Fig. 3A shows that the TRIPOD ordinal score focusing on validation and the RQS score focusing on study reporting are correlated (Pearson correlation coefficient 0.70). This reflects the importance the RQS places on study validation. However, both the TRIPOD score and RQS score were relatively independent of our assessment of study methodological limitations (Fig. 3B-C, Pearson correlation coefficients -0.12 and 0.13). Indeed, some studies with high TRIPOD and RQS scores had several technical limitations listed. For example, two studies with a TRIPOD score of 4 and the highest reported RQS scores (16 and 18 respectively) [14,15], had five and six identified methodological limitations respectively. In contrast, one study with a low TRIPOD score of 1b and a moderate RQS score (of 7) had just three pipeline technical limitations [18].

An illustrative example is given by three studies [14,15,23] that externally validated the landmark radiomic signature developed by Aerts and colleagues in 2014 [13]. However, subsequent work [111,129] has suggested that the prognostic value of the signature reflected the correlation of the signature with tumor volume, rather than reflecting underlying tumor heterogeneity. An important workflow step our review identified is the assessment of feature correlations and potential confounders (G). While the RQS recommends performing multivariable analysis and testing the benefit of the radiomics signature to a gold standard, it does not explicitly recommend testing for feature correlations or confounders.

Study quality depends not only on quality of reporting, but also on ensuring that features used are robust against potential problems. There is a raised recognition of the methodological issues that limit the potential utility of the radiomics concept, as shown by the increase in studies in this area (Fig. 2). However, we find that only 39 % of the patient outcome studies and 50 % of the biology studies we identified cite methodology papers. This suggests that there is still limited appreciation of the need to employ more rigorous radiomics workflows. The IBSI guidelines and RQS are aimed at addressing these issues. For example the IBSI reference manual gives recommendations for image processing techniques as well as suggesting standardized feature definitions, nomenclature, and guidelines for reporting [121]. The RQS rewards the use of test-retest approaches, multiple segmentation analyses, and the use of phantoms to resolve inter-scanner differences.

However, our review of limitations highlights further concerns, such as differing slice thickness or voxel size (C) and the specification of grey-level binning size (F). These are not included in RQS (only 58 % of studies in Table 1, Table 2 specified the grey-level binning method or size). The IBSI guidelines, the RQS and TRIPOD assessment schemes are important steps that should improve the technical quality of radiomics studies. However, they are not sufficient alone and review of the literature suggests a need to either update them to include more granular limitations or to use them alongside other assessment tools.

One result of the increase prevalence of studies investigating methodological limitations that would accelerate clinical translation would be the identification of a subset of robust features that should be used in outcome studies. Unfortunately, comparing results across studies is difficult. In addition to the risks to reliability listed in Table 3, the software used for feature extraction often uses different nomenclature (one of issues the IBSI addresses) and can calculate ostensibly similar features in different ways and with different parameter settings so that they are not comparable [122]. Software use varied greatly across all studies included in this review. Of the patient outcome and biology studies, 15 % did not specify the software used, 48 % used in-house developed software and just 37 % used free or commercial options. These numbers are similar for the methodology studies; 14 % did not specify the software used, 40 % used in-house developed software and 47 % used free or commercial options. Four of the patient outcome and biology studies did not specify the features in the final radiomic signature at all. The result is that there is no consensus on which particular features or feature signatures should be used for clinical studies. However, there are now increasing numbers of studies that employ the techniques used to determine which features are reliable. Table 4 and Supplementary Tables 2 and 3 list the remaining limitations for each clinical and biological study - 42 % of the assessed studies applied at least one of the suggested solutions to methodological limitations to increase feature robustness. Of these studies, 46 % used a test re-test dataset, 58 % used multiple segmentations and 4% tested CT model dependence.

A further important step in the radiomics workflow where community consensus would increase the comparability of studies is that of the optimal machine learning techniques that should be used to develop the resulting statistical models. We found that the top feature reduction technique used in all studies was univariable analysis (53 %) followed by LASSO (27 %). The most common modelling technique was logistic regression (39 %) followed by cox regression (34 %). 16 % of studies used random forest and 11 % SVM, both of which were highlighted as high performing by the methodology studies [118,120]. The techniques used in each study are listed in Supplementary Tables 2 and 3. Four outcome studies used multiple modelling techniques to determine which one performed best on their data; a recommended method as model performance is dataset-dependent [126]. Out of these four studies, the best performing classifiers were random forest [72] and Naïve Bayes [38,67]. One study did not reveal the best performing model [69].

The lack of consensus in how to address limitations to the reliability of radiomics features, or of a preferred way to conduct the subsequent statistical modelling, means there is still significant variability in approach, with each finely tuned to its own particular dataset. Progress along the imaging biomarker translation roadmap [12] is dependent on the development of reliable measures that can be used to test clinical hypotheses. These findings agree with those of previous authors [121,128] and show there is still an unmet need to move away from the current heterogeneous landscape to one that is more standardized. The validation of existing signatures in different datasets [14,15,23] discussed above is a vital part of this effort.

Lastly, in addition to the assessment of technical quality, radiomic signatures need to be evaluated for clinical relevance. It is important to test whether incorporating radiomic features into a clinical model improves performance over known prognostic or predictive factors. This need is well-recognized with 64 % of the studies in in Table 1 making its assessment. Future studies will be most impactful if they explicitly evaluate the clinical utility of a radiomic signature as part of data reporting.

In summary, use of the four different assessment tools allows us to draw three conclusions. Firstly, there is a high prevalence of methodological limitations among CT radiomics studies exploring the potential of the approach to guide personalized medicine. Secondly, there remains considerable variability in the approach to addressing these limitations, and that modelling approaches are likely tuned to specific datasets. Thirdly, different assessment tools provided complementary information, which taken together provided the greatest insight into how study data could be improved.

## 6 Future directions

Personalized medicine is of great potential benefit to patients, but this vision is dependent on the identification of stratification and predictive biomarkers [5]. Imaging biomarkers, derived from routinely acquired patient images, have enormous translational potential given the ubiquity of imaging in clinical workflows. Evaluation of the radiomics literature in NSCLC reveals the exponential rate of publication of new radiomics studies, which, in their conclusions, present a very positive view of the potential for radiomics to deliver this goal.

This review puts these findings in context for NSCLC, but the messages are likely to be generic to all cancer types. All published studies are at risk of translational hurdles due to technical and methodological issues. Importantly, some of these limitations are well recognized, well investigated and have solutions proposed that are beginning to be applied to clinical studies. In distinction, other limitations are poorly understood or researched, and so substantial barriers to translation remain. In addition, wider concerns surrounding over-fitting data and biological validation persist. Lastly, no single radiomic signature or methodological approach is used widely, so further work is required to identify candidates to take forward in larger multicenter studies.

The fact that all the radiomics studies identified in the NSCLC literature have some limitations should not infer that the published data and conclusions are incorrect; rather that risk exists in interpreting their findings at face value. Standardization issues, variability in methodology and a general lack of reporting hinders comparison of results across studies. Identifying limitations, by employing recognized assessment methodology tools, can help inform and educate design of future radiomics studies in NSCLC and beyond. This will improve study quality and expedite the translation of radiomic biomarkers as tools in personalized medicine.

## Declaration of Competing Interest

The authors declare no conflict of interest.
