# Supplementary material for: Radiomics as a personalized medicine tool in lung cancer: Separating the hope from the hype
Source: Lung Cancer. 2020 Aug;146:197–208. doi: 10.1016/j.lungcan.2020.05.028 (PMC7383235; doi:10.1016/j.lungcan.2020.05.028)
Supplement: Supplementary file 1 [file mmc1.docx]

Supplementary

# **Literature search**

*Search strategy*

Publications that report radiomics analyses on NSCLC data with the aim of predicting patient outcome were identified by searching the PubMed database using the key words “radiomics” and “lung cancer” or “NSCLC”. The search was conducted on the 08/01/2020 and no start date limit was used.

A second search was undertaken to find studies that addressed a methodological concern of radiomics. The PubMed database was searched using a combination of the following keys words (a) “radiomics” or “radiomics” and (b) “cancer” and (c) “standardization” or “reliable” or “impact of” or “improvement” or “repeatable” or “reproducible” or “repeatability” or “reproducibility” or “test–retest” or “variability” or “limitation” or “limitations” or “vulnerability” or “vulnerabilities” or “stability” or “stable” or “robustness” or “robust” or “quality” or “agreement” or “effect of”. The search was conducted on the 13/01/2020 and no start date limit was used.

*Search outcomes*

The results of the search for radiomics studies in lung cancer were screened by the title and abstract to find studies whose primary aim was either to create predictive radiomics models of clinical outcome or link radiomics to biology for NSCLC patients from CT images. Inclusion criteria were publications assessing outcomes of overall survival, metastases, treatment-induced toxicities or finding biological correlations. Studies using a modality other than CT, where the primary cancer was not NSCLC and review articles were not included in this step. 282 publications were found and after screening titles and abstracts based on the inclusion criteria, 116 publications remained. Exclusion criteria included CT studies not from planning CT, CBCT or diagnostic CT, if access to the article could not be gained, if the article was in a language other than English, if the study included deep learning as opposed to the traditional radiomics workflow discussed in this review, and studies predicting nodule malignancy. Studies of analysis reproducibility or methodology limitations were also excluded from this search, as they were included in the second evaluation. In all, 64 publications remained for analysis (Supplementary Figure 1). Included studies are summarized in Tables 1 and 2 and expanded in Supplementary Tables 2 and 3.

Supplementary Figure 1 Flow diagram for the patient outcome and biology radiomics studies in lung cancer search outcomes.


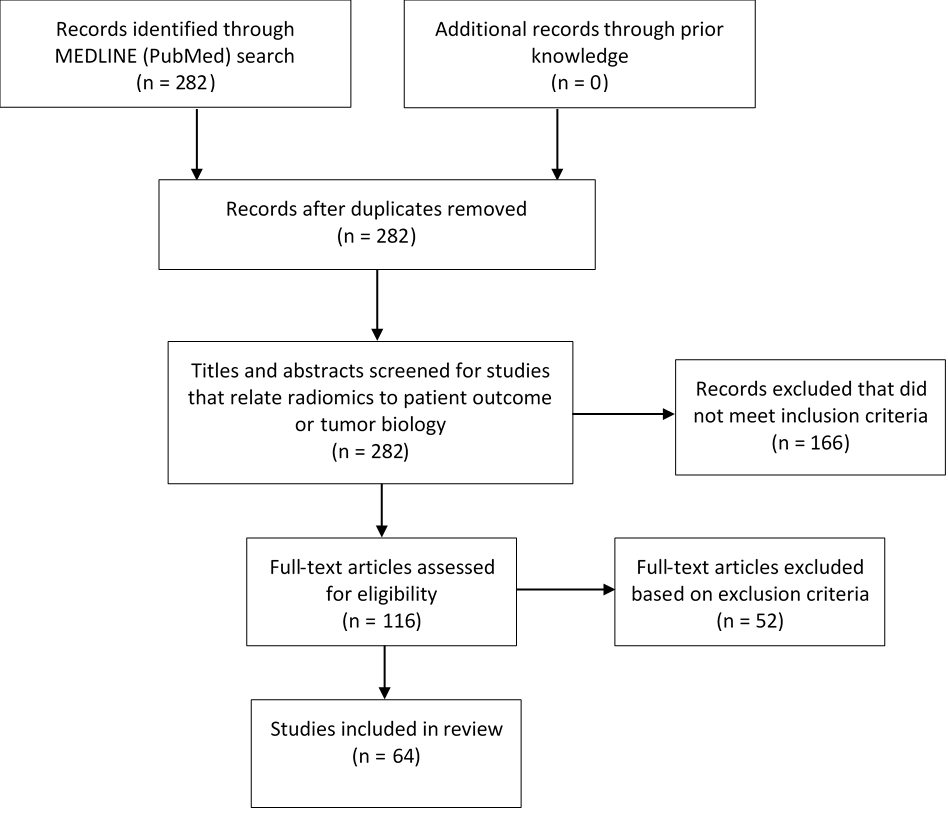


The results of the search for studies of radiomics limitations were screened by titles and abstract to identify studies whose primary aim was to address a radiomics-based methodological concern using human or phantom scans. 489 publications were found and a further 3 studies previously known to the authors but not returned by our search were added to the results. After applying screening by inclusion criteria 132 studies remained. The following exclusion criteria were then applied: the study data was not CT-based, the CT data was from a cancer other than NSCLC or not clearly specified, the study investigated variability in deep learning models rather than the traditional radiomics workflow, and the article was a review or report of a published public dataset, rather than original research. This approach left 42 studies for inclusion in this review (Supplementary Figure 2). Included studies are presented in Supplementary Table 1 and summarized in Table 3.

Supplementary Figure 1 Flow diagram for the methodological radiomics studies in lung cancer search outcomes.


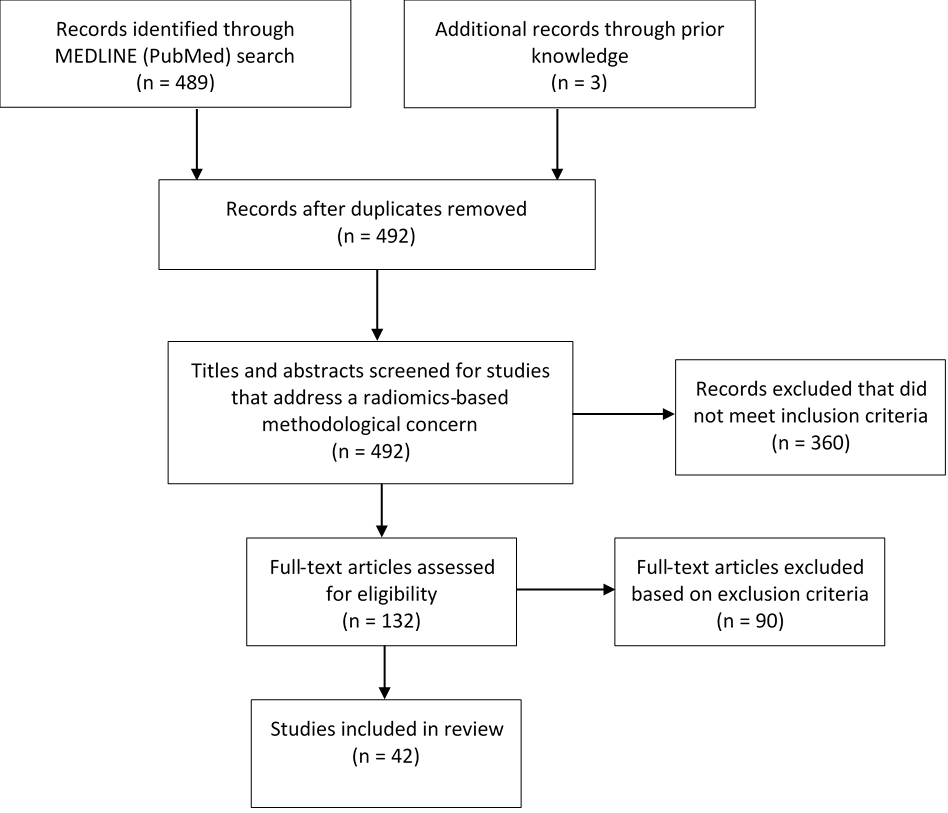


*Search constraints and limitations*

Conference abstracts were not included in the search. While this would have increased the number of studies included, abstracts had insufficient detail for our critical appraisal. Publication bias is a potential limitation of this review, since negative results are less likely to be published. This review included publications that investigated methodological concerns in the radiomics workflow for NSCLC and phantom CT scans and as such concerns that had been addressed in another cancer type or imaging modality were excluded from this analysis.

Supplementary Table 1 Radiomics methodological studies selected for inclusion.

Abbreviations: AIP, average intensity projection; CBCT, cone-beam CT; CCR, Credence Cartridge Radiomics phantom; HNSCC, head and neck squamous cell carcinoma; IBEX, imaging biomarker explorer; MPM, malignant pleural mesothelioma; NSCLC, non-small cell lung cancer; pCT, radiotherapy planning CT; ROI, region of interest.

| **Reference** | **Workflow stage(s)** | **Study data** | **Software** | **Features** | **Identified problems** | **Solution** |
| --- | --- | --- | --- | --- | --- | --- |
| Ger et al. 2018^1^ | Image acquisition.  Pre-processing. | Updated CCR phantom. 20 NSCLC and 30 HNSCC CT scans. | IBEX. | First order and texture. | CT scanner, protocol and slice thickness affect feature values. In general, resampling does not change feature values or remove feature correlations with slice thickness. | Correct for the CT manufacturer and model by scanning a phantom on each scanner. Control or limit the range of slice thickness in studies. |
| Fave et al. 2015^2^ | Image acquisition.  Test-retest. Volume dependence. | CCR phantom. 10 NSCLC CBCT test-retest scans. | IBEX. | First order and texture. | Features are more likely to be reproducible when the same CBCT scanner manufacturer and protocol are used. Increased motion reduces feature reproducibility. | A motion threshold of at most 10 mm, preferably 5 mm, increases feature reproducibility, as does excluding edges of the ROI. Texture features should not be compared across images acquired using different imaging protocols and CBCT manufacturers. |
| Lafata et al. 2018^3^ | Image acquisition. | Dynamic digital phantom simulation.  31 NSCLC free breathing CT scans, AIP and end of exhale 4DCT scans. | In-house Matlab. | Shape, first order and texture. | Image noise and motion affects feature reproducibility. | The end of exhale phase of a 4DCT is least affected by motion. |
| Du et al. 2019^4^ | Image acquisition. | 20 NSCLC 4DCT scans.  140 NSCLC 4DCT scans. | 3D Slicer. | Shape, first order and texture. | Motion affects feature reproducibility. | Assessing feature stability across all 4DCT phases can find features robust to motion which can improve the predictive performance of radiomic models. |
| Larue et al. 2017^5^ | Image acquisition. Pre-processing. | CCR phantom. | In-house. | First order and texture. | Feature values are different between CT scanners, even with similar acquisition protocols. Most radiomic features are affected by slice thickness. Choice of bin width influences feature values. The resampling method can induce variability in texture features. | Grey-level discretization could be optimized to improve prognostic value of features. Resampling decreases variability and reduces feature correlations with slice thickness. Cubic or linear interpolation induce less feature variability than nearest neighbour interpolation when resampling to 1x1x3mm3 voxels. |
| Mackin et al. 2015^6^ | Image acquisition. | CCR phantom. 20 NSCLC CT scans. | IBEX. | First order and texture. | Feature values are different between CT scanners. | Credentialing CT scanners could reduce variability across features measured on different scanners. CT scanners could be corrected for during data analysis. |
| Mackin et al. 2017^7^ | Image acquisition. Pre-processing. | CCR phantom. 8 NSCLC unconstructed CT scans. | IBEX. | Shape, first order and texture. | Variation in pixel size causes the intra-patient variability to be large relative to the inter-patient variability. | Feature variability due to differences in pixel size can be reduced through resampling and Butterworth low-pass filtering. |
| MacKin et al. 2018^8^ | Image acquisition. | CCR phantom. | IBEX. | First order and texture. | The impact of noise, i.e. tube current values, is more apparent for homogeneous materials than for textured. | Features are not substantially affected by variations in x-ray tube current. Tube current would not need to be harmonized across patients in a study. |
| Mahmood et al. 2017^9^ | Image acquisition. Image reconstruction. | Anthropomorphic phantom. | IBEX. | First order and texture. | Texture features are not reproducible across different CT scanners, even whilst using almost identical scanning parameters. | Robust correction factors need to be developed to reduce feature variability across CT scanners. |
| Midya et al. 2018^10^ | Image acquisition. Image reconstruction. | A uniform water phantom and an anthropomorphic phantom. A single abdominal CT scan. | In-house Matlab. | First order and texture. | CT scanner tube current, noise index and reconstruction technique influence feature reproducibility. | Only use features robust to changes in tube current, noise index and reconstruction technique. |
| Shafiq-ul-Hassan et al. 2017^11^ | Image acquisition. Image reconstruction. | CCR phantom. | In-house. | Shape, first order and texture. | Most texture features are reconstruction kernel dependent. | The variability in features due to different reconstruction kernels can be reduced by applying the Noise Power Spectrum peak frequency and ROI maximum intensity as correction factors. |
| Shafiq-ul-Hassan et al. 2017^12^ | Image acquisition. Pre-processing. | CCR phantom. | In-house. | Shape, first order and texture. | Features are dependent on voxel size and number of grey levels used for discretization. | Feature definitions can be normalized by voxel size and number of grey levels to reduce their dependency. |
| Yasaka et al. 2017^13^ | Image acquisition. Pre-processing. | CCR phantom. | TexRad. | First order. | Unfiltered and filtered features are variable across different CT scanners. | Feature variability due to different CT scanners needs to be taken into consideration. |
| Lu et al. 2016^14^ | Image acquisition. Image reconstruction. | 32 NSCLC CT scans. | Not specified. | Shape, first order and texture. | Changing reconstruction algorithm and slice thickness affects feature values. | Image acquisition techniques needs to be standardized. |
| Zhao et al. 2016^15^ | Image acquisition. Test re-test. Image reconstruction. | 31 NSCLC CT test-retest scans. | In-house. | Shape, first order and texture. | Features depend on the reconstruction algorithm used. | Features derived from images reconstructed with sharp and smooth algorithms should not be compared. |
| Li et al. 2018^16^ | Image acquisition.  Image reconstruction. | 51 NSCLC CT scans. | In-house Matlab. | Shape and texture. | Images with 1mm slice thickness give models with greater predictive performance than those with 5mm slice thickness. | Use thinner slice thickness to increase predictive performance of models. |
| Park et al. 2019^17^ | Image acquisition. | 100 NSCLC CT scans. | Not specified. | First order and texture. | Radiomic features are not reproducible across different slice thicknesses. | Reproducibility can be improved by converting images to 1mm slice thickness using a convolutional neural network-based super-resolution algorithm. |
| Kim et al. 2019^18^ | Image acquisition.  Image reconstruction. | Thoracic phantom. | Not specified. | First order and texture. | CT slice thickness, exposure setting and reconstruction algorithm affect radiomic features. | Image acquisition and reconstruction parameters need to be standardized to avoid variability. |
| Zhovannik et al. 2019^19^ | Image acquisition. | Phantom.  221 NSCLC pCT scans. | PyRadiomics. | First order and texture. | Radiomic features depend on scanner signal-to-noise ratio (exposure setting). | A correction algorithm can be modelled to make radiomic features reproducible across different signal-to-noise ratios. |
| Tunali et al. 2019^20^ | Image acquisition.  Segmentation. | 40 NSCLC CT scans.  32 NSCLC non-contrast enhanced CT scans.  212 NSCLC CT scans. | In-house C++. | First order and texture. | Radiomic features are not reproducible across multiple segmentations or different image acquisitions. | Use stable and reproducible radiomic features as a feature selection tool in the radiomics workflow. |
| Kakino et al. 2019^21^ | Image acquisition. | 269 NSCLC diagnostic delayed phase CT scans. | PyRadiomics. | First order and texture. | Not all features are reproducible across contrast enhanced and non-contrast enhanced CT images. This also depends on patient characteristics. | Do not combine contrast enhanced and non-contrast enhanced images in radiomics analysis. |
| Hepp et al. 2020^22^ | Image acquisition. | 69 NSCLC CT with simulated dose reduction. | PyRadiomics. | First order and texture. | Radiomic feature values differed when CT dose level was changed. | Differences in CT dose levels should be taken into account in radiomics studies. |
| Mahon et al. 2019^23^ | Image acquisition. | Gammex CT electron density phantom and Qasar body phantom.  135 NSCLC CT. | PyRadiomics. | Shape, first order and texture. | Variability in imaging protocols can induce variability in extracted radiomic features. | ComBat harmonization can harmonize radiomic features extracted from CT images using different imaging protocols. |
| Haga et al. 2018^24^ | Segmentation. | 40 NSCLC maximum exhale 4DCT scans. | In-house Matlab. | Shape, first order and texture. | Differences in tumor segmentation can cause different features to become significant in the feature selection stage. | Multiple segmentation analysis can reveal features that are robust to delineation uncertainties. For good predictions ROIs need to be contoured by a specialist, such as a radiation oncologist. |
| Huang et al. 2017^25^ | Segmentation. | 46 NSCLC CT scans. | In-house. | Shape, first order, texture and delta features. | Differences in tumor segmentation can lead to differences in a feature’s predictive power. | Multiple segmentation analysis should be done to find robust features. |
| Kalpathy-Cramer et al. 2016^26^ | Segmentation. | 40 NSCLC CT scans. Thoracic phantom. | Various. | Shape, first order and texture. | Some features are not robust to multiple segmentations. | Features need to be assessed for their robustness to segmentation and their usefulness in a predictive models. |
| Owens et al. 2018^27^ | Segmentation. | 10 NSCLC CT scans. | IBEX. | Shape, first order and texture. | Segmentation is time consuming and subject to inter-observer variability. | Semi-automatic segmentations performed by non-specialists can give segmentations comparable to those from clinicians. |
| Parmar et al. 2014^28^ | Segmentation. | 20 NSCLC CT scans. | In-house Matlab. | Shape, first order and texture. | Segmentation is time consuming and subject to inter-observer variability. | Semi-automatic segmentation led to a smaller range of feature values across observers than manual segmentation. |
| Pavic et al. 2018^29^ | Segmentation. | 11 NSCLC, 11 HNSCC, 11 MPM CT scans. | In-house Python. | Shape, first order and texture. | Features affected by delineation uncertainty are different between cancer types. Shape features are most affected across all tumor types. | Filtering the image increases the number of stable features. Multiple segmentation analysis should be performed to find robust features. Averaging texture matrices rather than merging results in more stable features with respect to segmentation. |
| Shafiq-ul-Hassan et al. 2018^30^ | Pre-processing. Volume dependence. | CCR phantom. 18 NSCLC CT scans. | Not specified. | First order and texture. | Some texture features are not stable across a different number of grey levels used for discretization or voxels in the ROI. | Normalization by the number of grey levels or number of voxels In the ROI made some features more reproducible. |
| Fave et al. 2016^31^ | Pre-processing. Volume dependence. | 107 NSCLC 4DCT end of exhale scans. | IBEX. | First order and texture. | Some features are entirely ROI volume dependent. Features tended to be more correlated with ROI volume after Butterworth smoothing. | Feature formulas can be corrected to remove ROI volume dependence. |
| Wang et al. 2019^32^ | Pre-processing.  Volume dependence. | 50 NSCLC CT scans. | Matlab open-source toolkit.^33^ | First order and texture. | Texture features may be sensitive to the number of grey levels used for discretization, the method of discretization and the use of an intensity threshold. | Discretization of 128 grey levels provides a set of reproducible texture features, regardless of discretization method. Thresholding the ROIs before feature extraction also improves reproducibility. |
| Welch et al. 2019^34^ | Volume dependence. | 421 NSCLC CT scans. | PyRadiomics. | Shape, first order and texture. | Features from a previously published radiomic signature were found to be correlated with tumor volume. | Features should be tested for multicollinearity using statistical analysis or by data perturbation. |
| Choi et al. 2018^35^ | Volume dependence. | 14 NSCLC free breathing pCT scans. | Not specified. | First order and texture. | Features may not be robust to variations in tumor size and may be correlated with the normal lung volume surrounding the tumor. | Simulations involving tumors of different sizes can reveal features robust to changes in tumor volume. |
| Larue et al. 2017^36^ | Test-retest. | 26 NSCLC CT scans test-retest. 20 NSCLC and 20 oesophageal 4DCT scans. 120 oesophageal CT scans. | In-house. | Shape, first order and texture. | Test-retest is not always available for the phenotype of interest. | A 4DCT dataset can be used to find robust features across phases, as an alternative to a test-retest dataset. |
| Van Timmeren et al. 2016^37^ | Test-retest. | 40 rectal cancer CT scans. 27 NSCLC CT scans. | Not specified. | Shape, first order and texture. | Test-retest results are not generalizable across different cancer types. | Test-retest analysis should be performed for each cancer type with controlled CT scanners and imaging protocols. |
| Zwanenburg et al. 2019^38^ | Test-retest. | 31 NSCLC and 19 HNSCC CT scans. | In-house Python. | Shape, first order and texture. | Test-retest is not always available for the phenotype of interest. | Image perturbation could be an alternative to test-retest, giving multiple images to compare features across. |
| Tanaka et al. 2019^39^ | Test-retest. | 14 NSCLC 4DCT scans.  14 NSCLC CT scans test-retest. | IBEX. | Shape, first order and texture. | Results from test-retest may not be generalizable to different CT protocols. | 4DCT could be used as an alternative to test-retest imaging. Finding robust features across phases around the end-of-exhale phase rather than all 10 phases prevents excessive dimension reduction. |
| Parmar et al. 2015^40^ | Modelling. | 464 NSCLC pCT scans (spiral thoracic CT with or without contrast). | In-house Matlab. | Shape, first order and texture. | Choice of classification method causes variation in a model’s predictive performance. | Particular combinations of feature selection and classification methods give classification models with high predictive performance. |
| Sun et al. 2018^41^ | Modelling. | 283 NSCLC pCT scans (spiral thoracic CT with or without contrast). | In-house Matlab. | Shape, first order and texture. | Statistical methods to predict overall survival differ in their predictive performance. | Particular combinations of feature selection and machine learning methods give survival models with high predictive performance. |
| Zhang et al. 2017^42^ | Modelling. | 112 NSCLC CT scans. | In-house Matlab. | First order and texture. | Endpoints, feature selection and classification methods affect predictive performance. | Sample sizes above 50 give better predictive performance. Subsampling data to add to the minority class increases predictive performance. |
| Haga et al. 2019^43^ | Modelling. | 40 NSCLC maximum exhale 4DCT scans.  29 NSCLC CT scans. | In-house Matlab. | Shape, first order and texture. | Feature normalization can affect predictive performance. | Performance of classification models can be improved by normalizing features, particularly z-score normalization. |

Supplementary Table 2 Radiomics studies in NSCLC, split into sections based on their investigated endpoint. The Data column specifies the total number of patients involved in the study, in brackets split by training and validation cohorts if applicable and specifying other cancer types of cohorts if applicable. Note: Studies marked with * are validation studies and their RQS score components refer to methodology based on the previous published data.

Abbreviations: AUC, area under the curve; CBCT, cone-beam CT; CI, concordance index; DFS, disease free survival; DM, distant metastasis; GRD, gross residual disease; H&N, head and neck; HR, hazard ratio; LR, local relapse; LRR, local regional recurrence; LR-RFS, loco-regional recurrence-free survival; OS, overall survival; pCR, pathological complete response; pCT, radiotherapy planning CT scan; PFS, progression free survival; RFS, recurrence free survival.

| **Reference** | **NSCLC stage** | **Data (training + validation)** | **Software** | **Radiomic features in final model** | **Result** | **Feature selection** | **Model building** | **TRIPOD** | **RQS (max 36)** | **Methodological limitations** | **Added value of radiomics to clinical model tested?** |
| --- | --- | --- | --- | --- | --- | --- | --- | --- | --- | --- | --- |
| **Overall survival** | | | | | | | | | | | |
| Aerts et al. 2014^44^ | 1-3b | 647 pCT  (422 + 225) | In-house MATLAB | Shape, first order and texture | CI=0.65 | Test re-test  Multiple segmentations  Univariable analysis | Cox regression | 3 | 19 | A, B, C, D, G | Yes |
| Van Timmeren et al. 2017^45^* | 1-4 | 252 pCT and CBCT  (102 + 56 + 94) | In-house MATLAB | Shape, first order and texture | CI=0.69, 0.61, 0.59 (pCT)  CI=0.66,0.63,0.59 (CBCT) | Validation of Aerts et al. 2014^44^ | Validation of Aerts et al. 2014^44^ | 4 | 16 | A, B, C, D, F, G | No |
| Grossman et al. 2017^46^* | 1-3 | 351 diagnostic CT  (262 + 89) | Not specified | Shape, first order and texture | CI=0.60 | Validation of Aerts et al. 2014^44^ | Validation of Aerts et al. 2014^44^ | 4 | 18 | A, B, C, D, G | Yes |
| Grossman et al. 2017^46^ | 1-3 | 351 diagnostic CT  (262 + 89) | Not specified | Not specified | CI=0.61 | mRMR  Stepwise selection | Cox regression | 3 | 9 | A, B, C, D, E, G, H | Yes |
| Yu et al. 2017^47^ | 1 | 442 diagnostic CT  (147 + 295) | IBEX | First order and texture | CI=0.64 | Multiple segmentations  Random survival forests  Correlation analysis  Correlation to tumor size  Univariable analysis | Cox regression | 3 | 15 | A, B, C | Yes |
| Chaddad et al. 2017^48^ | 1-3b | 315 pCT | In-house MATLAB | Shape and texture | Average AUC=0.70-0.76 | None performed | Random forest | 1b | 6 | A, B, C, D, E, G, H | Yes |
| Fave et al. 2017^49^ | 3 | 107 4DCT end of exhale, planning and CBCT | IBEX | Shape and texture | CI=0.672 | CT model dependence  Correlation to tumor volume  Stepwise selection | Cox regression | 1b | 7 | D, E, H | Yes |
| Li et al. 2017^50^ | 1-2a | 59 follow up CT | Definiens Developer | Texture | AUC=0.81 | Correlation analysis  PCA  Univariable analysis  Stepwise selection or backward stepwise selection | Cox regression | 1b | 6 | A, B, C, D, E, F, G, H | Yes |
| Li et al. 2017^51^ | 1-2a | 92 4DCT  Average-CT or 50% phase-CT images were used for analysis | Definiens Developer | Shape and first order | AUC=0.728 | Correlation analysis  Stepwise selection or backward stepwise selection | Cox regression | 1b | 6 | B, C, D, E, F, G, H | Yes |
| Tang et al. 2018^52^ | 1-3 | 290 staging CT  (114 + 176) | IBEX | Shape, first order and texture | CI=0.72 | Multiple segmentations  Clustering  Univariable analysis | Cox regression | 3 | 10 | A, B, C, D, F, G, H | No |
| Bianconi et al. 2018^53^ | 1-3 | 203 pCT | Not specified | Shape and texture | HR=1.06-1.48 | Univariable analysis | Kaplan-Meier | 1a | 1 | A, B, C, D, E, H | No |
| De Jong et al. 2018^54^* | 4 | 195 diagnostic CT | In-house MATLAB and CERR | Shape, first order and texture | CI=0.576 | Validation of Aerts et al. 2014^44^ | Validation of Aerts et al. 2014^44^ | 4 | 14 | A, B, C, D, F, G | Yes |
| Lee et al. 2018^55^ | 1-3 | 339 CT (type not defined, just pre-operative within 2 weeks before surgery) | In-house MATLAB | Shape, first order and texture | CI=0.772 | Univariable analysis  Stepwise selection  LASSO | Cox regression | 1b | 5 | A, B, C, D, E, G, H | Yes |
| He et al. 2018^56^ | 1-3 | 186 CT  (298 after oversampling (223 + 75)) type not defined | PyRadiomics | Not specified | AUC=0.9296 | None performed | Random forest | 2a | 1 | A, B, C, D, E, F, G, H | No |
| Starkov et al. 2018^57^ | 1 | 116 pCT | MATLAB Generalized Riesz-Wavelet Toolbox v 1.0 | Texture | High risk vs low risk median p-values=0.04–0.07 | LASSO | Kaplan-Meier | 1b | -5 | A, B, C, D, E, F, H | No |
| Yang et al. 2018^58^ | 1-4 | 371 CT  (239 + 132) | In-house MATLAB | First order and texture | CI=0.702 | Multiple segmentations  LASSO | Cox regression | 3 | 11 | A, B, C, D, F, G, H | Yes |
| Wang et al. 2019^59^ | 3 | 70 pre-treatment and 97 post treatment CT from 118 patients | Not specified | Texture | CI=0.743 | Multiple segmentations  Clustering  Random survival forest  Backward stepwise selection  Correlation analysis | Cox regression | 1b | 6 | B, C, D, F, H | No |
| Shi et al. 2019^60^ | 3 | 11 CBCT from 23 patients | IBEX | First order | HR=0.21 | Test re-test  Multiple segmentation  Correlation analysis | Kaplan-Meier | 1a | 4 | A, B, C, D | Yes |
| Van Timmeren et al. 2019^61^ | 1-4 | 337 pCT and 2154 CBCTs from 337 patients  (141 + 94 + 61 + 41) | In-house MATLAB | First order and texture | CI=0.59, 0.54, 0.57 | Correlation analysis  LASSO | Cox regression | 3 | 19 | B, C, D, E, G, H | Yes |
| Huang et al. 2019^62^ | 1-4 | 371 CT  (254 + 63 + 54) | In-house MATLAB | Shape, first order and texture | CI=0.621, 0.649 | Test re-test  LASSO | Cox regression | 2a | 4 | A, B, C, D, E, F, G | No |
| Franceschini et al. 2019^63^ | 1-2 | 102 4DCT (start of inspiration)  (70 + 32) | LIFEx | Shape and texture | AUC=0.85 | Univariable analysis  Elastic net Backward stepwise selection | Cox regression | 2a | 2 | C, D, E, G, H | No |
| **Local or metastatic recurrence** | | | | | | | | | | | |
| Coroller et al. 2015^64^ | 2-3 | 182 pCT  (98 + 84) | In-house MATLAB and CERR | First order and texture | CI=0.6 | mRMR  Univariable analysis  Stepwise selection | Cox regression | 2b | 13 | A, B, C, D, E, G, H | Yes |
| Mattonen et al. 2016^65^ | 1 | 45 follow-up CT | In-house MATLAB | First order and texture | AUC=0.85 | Stepwise selection | SVM | 1b | -2 | B, C, D, E, G, H | Yes |
| Huynh et al. 2016^66^ | 1-2 | 113 CT (free breathing) | In-house MATLAB and 3D Slicer | First order and texture | Median CI=0.67 | Test re-test  PCA  Univariable analysis | Cox regression | 1b | 6 | A, B, C, D, E, G | Yes |
| Huynh et al. 2017^67^ | 1-2a | 112 free breathing CT and AIP CT | In-house MATLAB and 3D Slicer | Shape, first order and texture | AIP radiomics CI=0.667  FB radiomics CI=0.601 | Test re-test  PCA  Univariable analysis  LASSO | Cox regression | 1b | 4 | A, B, C, E, G | Yes |
| Fave et al. 2017^49^ | 3 | 107 4DCT end of exhale, planning and CBCT | IBEX | Shape and texture | CI=0.632, 0.558 (DM, LRR) | Stepwise selection | Cox regression | 1b | 7 | D, E, H | Yes |
| Li et al. 2017^50^ | 1-2a | 59 follow up CT | Definiens Developer | Texture | AUC=0.80, 0.80 (RFS, LR-RFS) | Correlation analysis  PCA  Univariable analysis  Stepwise selection or backward stepwise selection | Cox regression | 1b | 6 | A, B, C, D, E, F, H | Yes |
| Li et al. 2017^51^ | 1-2a | 92 4DCT  Average-CT or 50% phase-CT images were used for analysis | Definiens Developer | Shape | AUC=0.747, 0.690 (RFS, LL-RFS) | Correlation analysis  Univariable analysis  Stepwise selection or backward stepwise selection | Cox regression | 1b | 6 | B, C, D, E, F, H | Yes |
| Dou et al. 2018^68^ | 2-3 | 200 pCT  (100 + 100) | PyRadiomics | Texture | CI=0.65 | Test re-test  mRMR  Stepwise selection | Cox regression | 2b | 16 | A, C, E, G | Yes |
| Ferreira Junior et al. 2018^69^ | 1-4 | 68 CT  (52 + 16) | IBEX | Shape and texture | AUC=0.75, 0.71  (lymph node metastasis, DM) | ReliefF | Naive Bayes, k -nearest neighbors and neural network | 2a | 9 | A, B, C, D, E, F, G, H | No |
| Yang et al. 2018^70^ | 1-3 | 159CT  (106 + 53) | PyRadiomics | Shape, first order and texture | AUC=0.856 | LASSO  Backward stepwise selection | Logistic regression | 2b | 13 | B, E, F, G, H | Yes |
| Zhong et al. 2018^71^ | 1-2 | 492 CT | MaZda | First order and texture | AUC=0.972 | Multiple segmentations  ReliefF  PCA | SVM | 1b | 3 | A, B, C, D, F, H, G | Yes |
| Lafata et al. 2019^72^ | 1 | 70 CT | In-house MATLAB | Texture | Maximum AUC=0.72, 0.83, 0.60 (recurrence, LR, non-LR) | Univariable analysis  Truncated singular value decomposition  LASSO | Logistic regression | 1b | 2 | A, B, C, D, E, F, G, H | No |
| Akinci D’Antonoli et al. 2019^73^ | 1-2b | 124 CT | Moddicom | Shape, first order and texture | AUC 0.731, 0.750 (LR, DM) | Univariable analysis  Stepwise selection | Cox regression | 1b | 13 | A, B, E, F, G, H | Yes |
| He et al. 2019^74^ | Not specified | 717CT  (423 + 294) | In-house MATLAB | First order and texture | CI=0.734 | Multiple segmentations  Correlation analysis  Univariable analysis  LASSO  Backward stepwise selection | Logistic regression | 2b | 13 | A, B, C, D, F, G, H | No |
| Xu et al. 2019^75^ | 3-4 | 132 CT  (106 + 26) | In-house MATLAB | Texture | AUC=0.642 | Test re-test  LASSO | Cox regression | 2a | 12 | B, C, D, E, G, H | No |
| Franceschini et al. 2019^63^ | 1-2 | 102 4DCT (start of inspiration)  (70 + 32) | LIFEx | Shape, first order and texture | AUC=0.73 | Backward stepwise selection | Logistic regression | 2a | 2 | C, D, E, G, H | No |
| Ferreira-Junior et al. 2019^76^ | 1-4 | 85 CT | IBEX | Shape, first order and texture | AUC=0.92, 0.84 (DM, nodal metastasis) | Univariable analysis  ReliefF | Neural network | 1b | -2 | A, B, C, G, H | No |
| Cong et al. 2019^77^ | 1a | 649 venous phase CT  (455 + 194) | Artificial Intelligence Kit | Shape, first order and texture | AUC=0.851 | Multiple segmentations  Univariable analysis  LASSO | Random forest | 2a | 14 | B, C, D, G, H | Yes |
| **Treatment response, disease-free or progression-free survival** | | | | | | | | | | | |
| Coroller et al. 2016^78^ | 2-3 | 127 pCT | In-house MATLAB and 3D Slicer | Shape, first order and texture | Median AUC=0.65, 0.61 (GRD, pCR) | Test re-test  PCA  Univariable analysis | Logistic regression | 1b | 7 | A, B, C, D, E, G | Yes |
| Huang et al. 2016^79^ | 1-2 | 282 CT (141 + 141) | In-house MATLAB | First order and texture | HR=2.09 | Multiple segmentations  LASSO | Cox regression | 2a | 13 | A, B, C, D, F, G, H | Yes |
| Song et al. 2016^80^ | 1-4 | 152 CT  (80 + 72) | Not specified | Texture | HR= 2.35, 2.75 | Univariable analysis | Cox regression | 2a | 4 | A, B, C, D, E, F, G, H | No |
| Coroller et al. 2017^81^ | 2-3 | 85 pCT | In-house MATLAB and 3D Slicer | Shape, first order and texture | Median AUC=0.68, =0.71 (pCR, GRD) | Test re-test  PCA  Univariable analysis | Random forest | 1b | 3 | A, B, C, D, E, G | Yes |
| Tunali et al. 2019^82^ | 3b-4 | 228 CT | In-house MATLAB and C++ | Texture | AUC=0.804 | Test re-test  Univariable analysais  Correlation to tumor volume  Backwards stepwise selection | Logistic regression | 1a | 5 | A, B, D, E | Yes |
| Franceschini et al. 2019^63^ | 1-2 | 102 4DCT (start of inspiration)  (70 + 32) | LIFEx | Texture | AUC=0.88 | Univariable analysis  Elastic net  Backward stepwise selection | Cox regression | 2a | 2 | C, D, E, G, H | No |
| **Lung toxicity** | | | | | | | | | | | |
| Moran et al. 2017^83^ | 1 | 14 diagnostic CT | Not specified | First order and texture | AUC=0.689-0.750 | Univariable analysis | Logistic regression | 1a | -2 | A, B, C, E, G, H | Yes |
| Krafft et al. 2018^84^ | Not specified | 192 50% 4DCT phase | In-house MATLAB | First order and texture | Average AUC=0.68 | LASSO | Logistic regression | 1b | 0 | A, E, G, H | Yes |
| **Staging** | | | | | | | | | | | |
| Yuan et al. 2018^85^ | 1 | 327 CT | Artificial intelligence kit | First order and texture | AUC=0.938 | Recursive feature elimination | SVM | 1b | 2 | A, B, C, F, G, H | No |
| Yang et al. 2019^86^ | 1-3 | 256 CT | PyRadiomics | First order and texture | AUC= 0.93 | LASSO | Logistic regression | 1b | -3 | B, C, E, F, G, H | No |

Supplementary Table 3 Radiomics studies in NSCLC with an aspect of biology as the endpoint. The Data column specifies the total number of patients involved in the study, in brackets split by training and validation cohorts if applicable and specifying other cancer types of cohorts if applicable.

Abbreviations: ADC, adenocarcinoma; AUC, area under the curve; CI, concordance index; EGFR, epidermal growth factor receptor; KRAS, Kirsten rat sarcoma viral oncogene homolog; LCC, large cell carcinoma; NOS, not otherwise specified; OR, odds ratio; SCC, squamous cell carcinoma.

| **Reference** | **Stage** | **Endpoint** | **Data (training + validation)** | **Software** | **Radiomic features in final model** | **Result** | **Feature selection** | **Model** | **TRIPOD** | **RQS (max 36)** | **Methodological limitations** |
| --- | --- | --- | --- | --- | --- | --- | --- | --- | --- | --- | --- |
| **Genomics** | | | | | | | | | | | |
| Aerts et al. 2016^87^ | Early stage | EGFR | 47 diagnostic CT and follow-up | Not specified | Shape and texture | AUC=0.74-0.91 | Coefficient of variation  Correlation analysis  Univariable analysis | Logistic regression | 1a | 2 | B, E, F |
| Rios Velazquez et al. 2017^88^ | 1-4 | EGFR, KRAS | 705 diagnostic CT  (353 + 352) | In-house plug in for 3D Slicer | Shape, first order and texture | AUC=0.69-0.80 | Test re-test  PCA  mRMR | Random forest | 3 | 14 | A, B, C, D, E, F |
| Mei et al. 2018^89^ | Not specified | EGFR | 296 CT | PyRadiomics | Texture | AUC=0.664 | Univariable analysis | Logistic regression | 1a | -2 | A, B, C, E, F, G, H |
| Digumarthy et al. 2019^90^ | Not specified | EGFR | 93 CT | TexRAD | First order | AUC=0.713 | Univariable analysis | Logistic regression | 1a | -1 | A, B, C, E, F, G, H |
| Jia et al. 2019^91^ | 1-4 | EGFR | 504 CT  (345 + 158) | Not specified | Shape, first order and texture | AUC=0.802 | Univariable analysis | Random forest | 2a | 5 | A, B, D, E, F, G, H |
| Li et al. 2019^92^ | 1-4 | EGFR subtypes (19Del and L858R) | 312 CT  (236 + 76) | In-house C++ | Shape and first order  skewness, Gabor’s  MTRVariance, Gabor’s PTREntropy, and sex were the most  important features for predicting 19Del  skewness, Gabor’s  MTRVariance, Gabor’s PTREntropy, and sex were the most  important features for predicting 19Del  skewness,  sphericity, sex, and smoking were the most important features  for predi cting L858R | AUC= 0.775-0.793 | Multiple segmentations  Univariable analysis  Stepwise selection | Logistic regression | 2b | 14 | B, C, F, G, H |
| Tu et al. 2019^93^ | 1-4 | EGFR | 404 CT  (243 + 161) | In-house MATLAB | First order and texture | AUC=0.775 | Multiple segmentations  Univariable analysis  Clustering  Backwards stepwise selection | Logistic regression | 2a | 13 | A, B, C, D, F, G, H |
| Yang et al. 2019^94^ | Not specified | EGFR | 467 CT  (306 + 161) | PyRadiomics | Shape, first order and texture | AUC=0.789 | Mean decrease impurity importance from random forest | Random forest | 2a | 13 | B, D, E, F, G, H |
| Wang et al. 2019^95^ | 1-2 | EGFR, TP53 | 61 CT  (41 + 20) | PyRadiomics | First order and texture | AUC=0.604, 0.586 | LASSO | SVM | 2a | 10 | B, C, E, F, G, H |
| Wang et al. 2019^95^ | 1-2 | Tumor mutation burden | 61 CT  (41 + 20) | PyRadiomics | Texture | AUC=0.606 | LASSO | SVM | 2a | 10 | B, C, E, F, G, H |
| **Signaling pathways** | | | | | | | | | | | |
| Grossman et al. 2017^46^ | 1-3 | Various | 351 CT  (262 + 89) | Not specified | Shape, first order and texture | AUC=0.62-0.72 | Clustering | Logistic regression | 3 | 9 | A, B, C, D, E, G, H |
| Bak et al. 2018^96^ | 1-4 | Various | 57 CT | In-house MATLAB | First order and texture | OR=0.08-23.94 | Univariable analysis | Logistic regression | 1a | -5 | B, C, E, F, G, H |
| **Histopathology** | | | | | | | | | | | |
| Patil et al. 2016^97^ | Not specified | ADC, LCC, SCC, NOS | 317 pCT | In-house MATLAB | Shape, first order and texture | 88% accuracy | None | SVM | 1b | 3 | A, B, C, D, E, G, H |
| Wu et al. 2016^98^ | 1-4 | ADC, SCC | 350 pCT  (198 + 152) | In-house MATLAB | First order and texture | AUC=0.72 | Correlation analysis  Univariable analysis | Random forest, naive Bayes, and k-nearest neighbors | 3 | 13 | A, B, C, D, E, G, H |
| Ferreira Junior et al. 2018^69^ | 1-4 | ADC, SCC | 68 CT  (52 + 16) | IBEX | Not specified | AUC=0.81 | ReliefF | Naive Bayes and k-nearest neighbors and neural network | 2a | 6 | A, B, C, D, E, G, H |
| Zhu et al. 2018^99^ | Not specified | ADC, SCC | 129 CT  (81 + 48) | In-house MATLAB | First order and texture | AUC=0.893 | Multiple segmentations  LASSO | Logistic regression | 2a | 11 | A, B, C, D, F, G, H |
| Digumarthy et al. 2019^90^ | Not specified | ADC, SCC | 93 CT | TexRAD | First order | AUC=0.744 | Univariable analysis | Logistic regression | 1a | -1 | A, B, C, E, F, G, H |
| E et al. 2019^100^ | Not specified | ADC, SCC, SCLC | 229 CT | In-house MATLAB | Shape, first order and texture | AUC=0.657-0.875 | Test re-test  Clustering  mRMR  Incremental forward search | Naive Bayes, logistic regression and random forest | 1b | 5 | B, C, E, F, G |
| Ferreira-Junior et al. 2019^76^ | 1-4 | ADC, SCC | 85 CT | IBEX | Shape, first order, texture | AUC=0.88 | Univariable analysis  ReliefF | Neural network | 1b | -2 | A, B, C, G, H |
| Liu et al. 2019^101^ | Not specified | ADC, LCC, SCC, NOS | 349 CT  (278 + 71) | Not specified | Not specified | AUC=0.86 | l2,1-norm minimization | SVM | 3 | 4 | A, B, C, D, E, F, G, H |
| Zhou et al. 2018^102^ | 1-4 | Ki-67 | 110 CT | 3D Slicer | Shape and texture | AUC=0.61-0.77 | Univariable analysis  Backwards stepwise selection | Logistic regression | 1a | 11 | B, D, E, F, G, H |
| Gu et al. 2019^103^ | Not specified | Ki-67 | 245 CT | MaZda | First order and texture | AUC=0.776 | Feature selection algorithm based on random forest | Logistic regression, linear discriminant analysis, classification tree and regression tree, k-neighbour clustering, SVM and random forest | 1b | -2 | A, B, C, D, E, F, G, H |
| Song et al. 2017^104^ | 1-3 | Micropapillary pattern | 339 CT | Not specified | First order | AUC=0.751 | Multiple segmentation  Univariable analysis  Stepwise selection | Logistic regression | 1b | 8 | B, C, D, F, G, H |
| Chen et al. 2018^105^ | Not specified | Degree of differentiation | 487 CT  (303 + 184) | In-house MATLAB | First order and texture | AUC = 0.782 | Univariable analysis  mRMR  Backwards stepwise selection | Logistic regression | 2b | 3 | A, B, C, D, E, F, G, H |
| She et al. 2018^106^ | Not specified | Invasive vs non-invasive adenocarcinoma | 402 CT(207 + 195) | In-house Python | Shape, first order and texture | AUC=0.89 | LASSO | Logistic regression | 2b | 8 | A, B, C, D, E, F, G, H |
| Yang et al. 2019^107^ | Not specified | Invasive vs non-invasive adenocarcinoma | 192 CT  (116 + 76) | Artificial intelligence kit | First order and texture | AUC=0.77 | Multiple segmentations  Correlation analysis  LASSO | Logistic regression | 2a | 13 | B, C, D, F, G, H |

Supplementary Table 4 Transparent Reporting of a multivariable prediction model for Individual Prognosis Or Diagnosis (TRIPOD) analysis types.^108^

| **Analysis Type** | **Description** |
| --- | --- |
| Type 1a | Development of a prediction model where predictive performance is then directly evaluated using exactly the same data (apparent performance). |
| Type 1b | Development of a prediction model using the entire data set, but then using resampling (e.g. bootstrapping or cross-validation) techniques to evaluate the performance and optimism of the developed model. |
| Type 2a | The data are randomly split into two groups: one to develop the prediction model, and one to evaluate its predictive performance. |
| Type 2b | The data are non-randomly split (e.g. by location or time) into two groups: one to develop the prediction model and one to evaluate its predictive performance. |
| Type 3 | Development of a prediction model using one data set and an evaluation of its performance on separate data (e.g. from a different study) |
| Type 4 | The evaluation of the predictive performance of an existing (published) prediction model on separate data. |

Supplementary Table 5 The radiomics quality score (RQS) scoring criteria developed by Lambin et al.^109^

|  | Criteria | Points |
| --- | --- | --- |
| 1 | Image protocol quality – well-documented image protocols (e.g., contrast, slice thickness, energy, etc.) and/or usage of public image protocols allow reproducibility/ replicability | +1 (if protocols are well-documented)  +1 (if public protocol is used) |
| 2 | Multiple segmentations – possible actions are: segmentation by different physicians/ algorithms/software, perturbing segmentations by (random) noise, segmentation at different breathing cycles. Analyze feature robustness to segmentation variabilities | +1 |
| 3 | Phantom study on all scanners – detect inter-scanner differences and vendor-dependent features. Analyze feature robustness to these sources of variability | +1 |
| 4 | Imaging at multiple time points – collect individuals’ images at additional time points. Analyze feature robustness to temporal variabilities (e.g., organ movement, organ expansion/shrinkage). | +1 |
| 5 | Feature reduction or adjustment for multiple testing – decreases the risk of overfitting. Overfitting is inevitable if the number of features exceeds the number of samples. Consider feature robustness when selecting features | +3 (if neither measure is implemented)  +3 (if either measure is implemented) |
| 6 | Multivariable analysis with non radiomic features (e.g., EGFR mutation) – is expected to provide a more holistic model. Permits correlating/inferencing between radiomics and non radiomics features | +1 |
| 7 | Detect and discuss biological correlates – demonstration of phenotypic differences (possibly associated with underlying gene–protein expression patterns) deepens understanding of radiomics and biology | +1 |
| 8 | Cut-off analyses – determine risk groups by either the median, a previously published cut-off or report a continuous risk variable. Reduces the risk of reporting overly optimistic results | +1 |
| 9 | Discrimination statistics – report discrimination statistics (e.g., C-statistic, ROC curve, AUC) and their statistical significance (e.g., p-values, confidence intervals). One can also apply resampling method (e.g., bootstrapping, cross-validation) | +1 (if a discrimination statistic and its statistical significance are reported)  +1 (if also an resampling method technique is applied) |
| 10 | Calibration statistics – report calibration statistics (e.g., Calibration-in-the-large/slope, calibration plots) and their statistical significance (e.g., p-values, confidence intervals). One can also apply resampling method (e.g., bootstrapping, cross-validation) | +1 (if a calibration statistic and its statistical significance are reported)  +1 (if also an resampling method technique is applied) |
| 11 | Prospective study registered in a trial database – provides the highest level of evidence supporting the clinical validity and usefulness of the radiomics biomarker | +7 (for prospective validation of a radiomics signature in an appropriate trial) |
| 12 | Validation – the validation is performed without retraining and without adaptation of the cut-off value, provides crucial information with regard to credible clinical performance | -5 (if validation is missing)  +2 (if validation is based on a dataset from the same institute)  +3 (if validation is based on a dataset from another institute)  +4 (if validation is based on two datasets from two distinct institutes)  +4 (if the study validates a previously published signature)  +5 (if validation is based on three or more datasets from distinct institutes)  *Datasets should be of comparable size and should have at least 10 events per model feature. |
| 13 | Comparison to ‘gold standard’ – assess the extent to which the model agrees with/is superior to the current ‘gold standard’ method (e.g., TNM-staging for survival prediction). This comparison shows the added value of radiomics | +2 |
| 14 | Potential clinical utility – report on the current and potential application of the model in a clinical setting (e.g., decision curve analysis) | +2 |
| 15 | Cost-effectiveness analysis – report on the cost-effectiveness of the clinical application (e.g., quality adjusted life years generated) | +1 |
| 16 | Open science and data – make code and data publicly available. Open science facilitates knowledge transfer and reproducibility of the study | +1 (if scans are open source)  +1 (if region of interest segmentations are open source)  +1 (if code is open source)  +1 (if radiomics features are calculated on a set of representative ROIs and the calculated features + representative ROIs are open source |
|  | Total points (36 = 100%) |  |

# **References**

1. Ger RB, Zhou S, Chi PCM, et al. Comprehensive Investigation on Controlling for CT Imaging Variabilities in Radiomics Studies. *Sci Rep*. 2018;8(1):13047. doi:10.1038/s41598-018-31509-z

2. Fave X, Mackin D, Yang J, et al. Can radiomics features be reproducibly measured from CBCT images for patients with non-small cell lung cancer? *Med Phys*. 2015;42(12):6784-6797. doi:10.1118/1.4934826

3. Lafata K, Cai J, Wang C, Hong J, Kelsey CR, Yin FF. Spatialoral variability of radiomic features and its effect on the classification of lung cancer histology. *Phys Med Biol*. 2018;63(22):225003. doi:10.1088/1361-6560/aae56a

4. Du Q, Baine M, Bavitz K, et al. Radiomic feature stability across 4D respiratory phases and its impact on lung tumor prognosis prediction. *PLoS One*. 2019;14(5):e0216480. doi:10.1371/journal.pone.0216480

5. Larue RTHM, van Timmeren JE, de Jong EEC, et al. Influence of gray level discretization on radiomic feature stability for different CT scanners, tube currents and slice thicknesses: a comprehensive phantom study. *Acta Oncol (Madr)*. 2017;56(11):1544-1553. doi:10.1080/0284186X.2017.1351624

6. Mackin D, Fave X, Zhang L, et al. Measuring computed tomography scanner variability of radiomics features. *Invest Radiol*. 2015;50(11):757-765. doi:10.1097/RLI.0000000000000180

7. Mackin D, Fave X, Zhang L, et al. Harmonizing the pixel size in retrospective computed tomography radiomics studies. Tian J, ed. *PLoS One*. 2017;12(9):e0178524. doi:10.1371/journal.pone.0178524

8. Mackin D, Ger R, Dodge C, et al. Effect of tube current on computed tomography radiomic features. *Sci Rep*. 2018;8(1):2354. doi:10.1038/s41598-018-20713-6

9. Mahmood U, Apte AP, Deasy JO, Schmidtlein CR, Shukla-Dave A. Investigating the robustness neighborhood gray tone difference matrix and gray level co-occurrence matrix radiomic features on clinical computed tomography systems using anthropomorphic phantoms: Evidence from a multivendor study. *J Comput Assist Tomogr*. 2017;41(6):995-1001. doi:10.1097/RCT.0000000000000632

10. Midya A, Chakraborty J, Gönen M, Do RKG, Simpson AL. Influence of CT acquisition and reconstruction parameters on radiomic feature reproducibility. *J Med Imaging*. 2018;5(01):1. doi:10.1117/1.jmi.5.1.011020

11. Shafiq-ul-Hassan M, Zhang GG, Hunt DC, et al. Accounting for reconstruction kernel-induced variability in CT radiomic features using noise power spectra. *J Med Imaging*. 2017;5(01):1. doi:10.1117/1.jmi.5.1.011013

12. Shafiq-Ul-Hassan M, Zhang GG, Latifi K, et al. Intrinsic dependencies of CT radiomic features on voxel size and number of gray levels. *Med Phys*. 2017;44(3):1050-1062. doi:10.1002/mp.12123

13. Yasaka K, Akai H, Mackin D, et al. Precision of quantitative computed tomography texture analysis using image filtering. *Med (United States)*. 2017;96(21):e6993. doi:10.1097/MD.0000000000006993

14. Lu L, Ehmke RC, Schwartz LH, Zhao B. Assessing agreement between radiomic features computed for multiple CT imaging settings. *PLoS One*. 2016;11(12):e0166550. doi:10.1371/journal.pone.0166550

15. Zhao B, Tan Y, Tsai WY, et al. Reproducibility of radiomics for deciphering tumor phenotype with imaging. *Sci Rep*. 2016;6(1):23428. doi:10.1038/srep23428

16. Li Y, Lu L, Xiao M, et al. CT Slice Thickness and Convolution Kernel Affect Performance of a Radiomic Model for Predicting EGFR Status in Non-Small Cell Lung Cancer: A Preliminary Study. *Sci Rep*. 2018;8(1):17913. doi:10.1038/s41598-018-36421-0

17. Park S, Lee SM, Do KH, et al. Deep learning algorithm for reducing ct slice thickness: Effect on reproducibility of radiomic features in lung cancer. *Korean J Radiol*. 2019;20(10):1431-1440. doi:10.3348/kjr.2019.0212

18. Kim YJ, Lee HJ, Kim KG, Lee SH. The Effect of CT Scan Parameters on the Measurement of CT Radiomic Features: A Lung Nodule Phantom Study. *Comput Math Methods Med*. 2019;2019:8790694. doi:10.1155/2019/8790694

19. Zhovannik I, Bussink J, Traverso A, et al. Learning from scanners: Bias reduction and feature correction in radiomics. *Clin Transl Radiat Oncol*. 2019;19:33-38. doi:10.1016/j.ctro.2019.07.003

20. Tunali I, Hall LO, Napel S, et al. Stability and reproducibility of computed tomography radiomic features extracted from peritumoral regions of lung cancer lesions. *Med Phys*. 2019;46(11):5075-5085. doi:10.1002/mp.13808

21. Kakino R, Nakamura M, Mitsuyoshi T, et al. Comparison of radiomic features in diagnostic CT images with and without contrast enhancement in the delayed phase for NSCLC patients. *Phys Med*. 2020;69:176-182. doi:10.1016/j.ejmp.2019.12.019

22. Hepp T, Othman A, Liebgott A, Kim JH, Pfannenberg C, Gatidis S. Effects of simulated dose variation on contrast-enhanced CT-based radiomic analysis for Non-Small Cell Lung Cancer. *Eur J Radiol*. 2020;124:108804. doi:10.1016/j.ejrad.2019.108804

23. Mahon RN, Ghita M, Hugo GD, Weiss E. ComBat harmonization for radiomic features in independent phantom and lung cancer patient computed tomography datasets. *Phys Med Biol*. 2020;65(1). doi:10.1088/1361-6560/ab6177

24. Haga A, Takahashi W, Aoki S, et al. Classification of early stage non-small cell lung cancers on computed tomographic images into histological types using radiomic features: interobserver delineation variability analysis. *Radiol Phys Technol*. 2018;11(1):27-35. doi:10.1007/s12194-017-0433-2

25. Huang Q, Lu L, Dercle L, et al. Interobserver variability in tumor contouring affects the use of radiomics to predict mutational status. *J Med Imaging*. 2017;5(01):1. doi:10.1117/1.jmi.5.1.011005

26. Kalpathy-Cramer J, Mamomov A, Zhao B, et al. Radiomics of Lung Nodules: A Multi-Institutional Study of Robustness and Agreement of Quantitative Imaging Features. *Tomography*. 2016;2(4):430-437. doi:10.18383/j.tom.2016.00235

27. Owens CA, Peterson CB, Tang C, et al. Lung tumor segmentation methods: Impact on the uncertainty of radiomics features for non-small cell lung cancer. Fan Y, ed. *PLoS One*. 2018;13(10):1-22. doi:10.1371/journal.pone.0205003

28. Parmar C, Velazquez ER, Leijenaar R, et al. Robust radiomics feature quantification using semiautomatic volumetric segmentation. Woloschak GE, ed. *PLoS One*. 2014;9(7):e102107. doi:10.1371/journal.pone.0102107

29. Pavic M, Bogowicz M, Wurms X, et al. Influence of inter-observer delineation variability on radiomics stability in different tumor sites. *Acta Oncol (Madr)*. 2018;57(8):1070-1074. doi:10.1080/0284186X.2018.1445283

30. Shafiq-Ul-Hassan M, Latifi K, Zhang G, Ullah G, Gillies R, Moros E. Voxel size and gray level normalization of CT radiomic features in lung cancer. *Sci Rep*. 2018;8(1):10545. doi:10.1038/s41598-018-28895-9

31. Fave X, Zhang L, Yang J, et al. Impact of image preprocessing on the volume dependence and prognostic potential of radiomics features in non-small cell lung cancer. *Transl Cancer Res*. 2016;5(4):349-363. doi:10.21037/tcr.2016.07.11

32. Wang HYC, Donovan EM, Nisbet A, et al. The stability of imaging biomarkers in radiomics: A framework for evaluation. *Phys Med Biol*. 2019;64(16):165012. doi:10.1088/1361-6560/ab23a7

33. Vallières M, Freeman CR, Skamene SR, et al. A radiomics model from joint FDG-PET and MRI texture features for the prediction of lung metastases in soft-tissue sarcomas of the extremities. *Phys Med Biol*. 2015;60(14):5471-5496. doi:10.1088/0031-9155/60/14/5471

34. Welch ML, McIntosh C, Haibe-Kains B, et al. Vulnerabilities of radiomic signature development: The need for safeguards. *Radiother Oncol*. 2019;130:2-9. doi:10.1016/j.radonc.2018.10.027

35. Choi W, Riyahi S, Kligerman SJ, Liu C-J, Mechalakos JG, Lu W. Technical Note: Identification of CT Texture Features Robust to Tumor Size Variations for Normal Lung Texture Analysis. *Int J Med Physics, Clin Eng Radiat Oncol*. 2018;07(03):330-338. doi:10.4236/ijmpcero.2018.73027

36. Larue RTHM, Van De Voorde L, van Timmeren JE, et al. 4DCT imaging to assess radiomics feature stability: An investigation for thoracic cancers. *Radiother Oncol*. 2017;125(1):147-153. doi:10.1016/j.radonc.2017.07.023

37. Van Timmeren JE, Leijenaar RTH, van Elmpt W, et al. Test–Retest Data for Radiomics Feature Stability Analysis: Generalizable or Study-Specific? *Tomography*. 2016;2(4):361-365. doi:10.18383/j.tom.2016.00208

38. Zwanenburg A, Leger S, Agolli L, et al. Assessing robustness of radiomic features by image perturbation. *Sci Rep*. 2019;9(1):614. doi:10.1038/s41598-018-36938-4

39. Tanaka S, Kadoya N, Kajikawa T, et al. Investigation of thoracic four-dimensional CT-based dimension reduction technique for extracting the robust radiomic features. *Phys Medica*. 2019;58:141-148. doi:10.1016/j.ejmp.2019.02.009

40. Parmar C, Grossmann P, Bussink J, Lambin P, Aerts HJWL. Machine Learning methods for Quantitative Radiomic Biomarkers. *Sci Rep*. 2015;5(1):13087. doi:10.1038/srep13087

41. Sun W, Jiang M, Dang J, Chang P, Yin FF. Effect of machine learning methods on predicting NSCLC overall survival time based on Radiomics analysis. *Radiat Oncol*. 2018;13(1):197. doi:10.1186/s13014-018-1140-9

42. Zhang Y, Oikonomou A, Wong A, Haider MA, Khalvati F. Radiomics-based Prognosis Analysis for Non-Small Cell Lung Cancer. *Sci Rep*. 2017;7(1):46349. doi:10.1038/srep46349

43. Haga A, Takahashi W, Aoki S, et al. Standardization of imaging features for radiomics analysis. *J Med Invest*. 2019;66(12):35-37. doi:10.2152/jmi.66.35

44. Aerts HJWL, Velazquez ER, Leijenaar RTH, et al. Decoding tumour phenotype by noninvasive imaging using a quantitative radiomics approach. *Nat Commun*. 2014;5(4006):4006. doi:10.1038/ncomms5006

45. van Timmeren JE, Leijenaar RTH, van Elmpt W, et al. Survival prediction of non-small cell lung cancer patients using radiomics analyses of cone-beam CT images. *Radiother Oncol*. 2017;123(3):363-369. doi:10.1016/j.radonc.2017.04.016

46. Grossmann P, Stringfield O, El-Hachem N, et al. Defining the biological basis of radiomic phenotypes in lung cancer. *Elife*. 2017;6:1-22. doi:10.7554/eLife.23421

47. Yu W, Tang C, Hobbs BP, et al. Development and Validation of a Predictive Radiomics Model for Clinical Outcomes in Stage I Non-small Cell Lung Cancer. *Int J Radiat Oncol Biol Phys*. 2017;102(4):1090-1097. doi:10.1016/j.ijrobp.2017.10.046

48. Chaddad A, Desrosiers C, Toews M, Abdulkarim B. Predicting survival time of lung cancer patients using radiomic analysis. *Oncotarget*. 2017;8(61):104393-104407. doi:10.18632/oncotarget.22251

49. Fave X, Zhang L, Yang J, et al. Delta-radiomics features for the prediction of patient outcomes in non-small cell lung cancer. *Sci Rep*. 2017;7(1):588. doi:10.1038/s41598-017-00665-z

50. Li Q, Kim J, Balagurunathan Y, et al. CT imaging features associated with recurrence in non-small cell lung cancer patients after stereotactic body radiotherapy. *Radiat Oncol*. 2017;12(1):158. doi:10.1186/s13014-017-0892-y

51. Li Q, Kim J, Balagurunathan Y, et al. Imaging features from pretreatment CT scans are associated with clinical outcomes in nonsmall-cell lung cancer patients treated with stereotactic body radiotherapy. *Med Phys*. 2017;44(8):4341-4349. doi:10.1002/mp.12309

52. Tang C, Hobbs B, Amer A, et al. Development of an Immune-Pathology Informed Radiomics Model for Non-Small Cell Lung Cancer. *Sci Rep*. 2018;8(1):1922. doi:10.1038/s41598-018-20471-5

53. Bianconi F, Fravolini ML, Bello-Cerezo R, Minestrini M, Scialpi M, Palumbo B. Evaluation of shape and textural features from CT as prognostic biomarkers in non-small cell lung cancer. *Anticancer Res*. 2018;38(4):2155-2160. doi:10.21873/anticanres.12456

54. de Jong EEC, van Elmpt W, Rizzo S, et al. Applicability of a prognostic CT-based radiomic signature model trained on stage I-III non-small cell lung cancer in stage IV non-small cell lung cancer. *Lung Cancer*. 2018;124:6-11. doi:10.1016/j.lungcan.2018.07.023

55. Lee G, Park H, Sohn I, et al. Comprehensive Computed Tomography Radiomics Analysis of Lung Adenocarcinoma for Prognostication. *Oncologist*. 2018;23(7):806-813. doi:10.1634/theoncologist.2017-0538

56. He B, Zhao W, Pi JY, et al. A biomarker basing on radiomics for the prediction of overall survival in non-small cell lung cancer patients. *Respir Res*. 2018;19(1):199. doi:10.1186/s12931-018-0887-8

57. Starkov P, Aguilera TA, Golden DI, et al. The use of texture-based radiomics CT analysis to predict outcomes in early-stage non-small cell lung cancer treated with stereotactic ablative radiotherapy. *Br J Radiol*. 2019;92(1094):20180228. doi:10.1259/bjr.20180228

58. Yang L, Yang J, Zhou X, et al. Development of a radiomics nomogram based on the 2D and 3D CT features to predict the survival of non-small cell lung cancer patients. *Eur Radiol*. 2019;29(5):2196-2206. doi:10.1007/s00330-018-5770-y

59. Wang L, Dong T, Xin B, et al. Integrative nomogram of CT imaging, clinical, and hematological features for survival prediction of patients with locally advanced non-small cell lung cancer. *Eur Radiol*. 2019;29(6):2958-2967. doi:10.1007/s00330-018-5949-2

60. Shi L, Rong Y, Daly M, et al. Cone-beam computed tomography-based delta-radiomics for early response assessment in radiotherapy for locally advanced lung cancer. *Phys Med Biol*. 2019;65(1):15009. doi:10.1088/1361-6560/ab3247

61. van Timmeren JE, van Elmpt W, Leijenaar RTH, et al. Longitudinal radiomics of cone-beam CT images from non-small cell lung cancer patients: Evaluation of the added prognostic value for overall survival and locoregional recurrence. *Radiother Oncol*. 2019;136:78-85. doi:10.1016/j.radonc.2019.03.032

62. Huang L, Chen J, Hu W, et al. Assessment of a Radiomic Signature Developed in a General NSCLC Cohort for Predicting Overall Survival of ALK-Positive Patients With Different Treatment Types. *Clin Lung Cancer*. 2019;20(6):e638-e651. doi:10.1016/j.cllc.2019.05.005

63. Franceschini D, Cozzi L, De Rose F, et al. A radiomic approach to predicting nodal relapse and disease-specific survival in patients treated with stereotactic body radiation therapy for early-stage non-small cell lung cancer. *Strahlentherapie und Onkol*. November 2019. doi:10.1007/s00066-019-01542-6

64. Coroller TP, Grossmann P, Hou Y, et al. CT-based radiomic signature predicts distant metastasis in lung adenocarcinoma. *Radiother Oncol*. 2015;114(3):345-350. doi:10.1016/j.radonc.2015.02.015

65. Mattonen SA, Palma DA, Johnson C, et al. Detection of Local Cancer Recurrence after Stereotactic Ablative Radiation Therapy for Lung Cancer: Physician Performance Versus Radiomic Assessment. *Int J Radiat Oncol Biol Phys*. 2016;94(5):1121-1128. doi:10.1016/j.ijrobp.2015.12.369

66. Huynh E, Coroller TP, Narayan V, et al. CT-based radiomic analysis of stereotactic body radiation therapy patients with lung cancer. *Radiother Oncol*. 2016;120(2):258-266. doi:10.1016/j.radonc.2016.05.024

67. Huynh E, Coroller TP, Narayan V, et al. Associations of radiomic data extracted from static and respiratory-gated CT scans with disease recurrence in lung cancer patients treated with SBRT. *PLoS One*. 2017;12(1):e0169172. doi:10.1371/journal.pone.0169172

68. Dou TH, Coroller TP, van Griethuysen JJM, Mak RH, Aerts HJWL. Peritumoral radiomics features predict distant metastasis in locally advanced NSCLC. *PLoS One*. 2018;13(11):e0206108. doi:10.1371/journal.pone.0206108

69. Ferreira Junior JR, Koenigkam-Santos M, Cipriano FEG, Fabro AT, Azevedo-Marques PM de. Radiomics-based features for pattern recognition of lung cancer histopathology and metastases. *Comput Methods Programs Biomed*. 2018;159:23-30. doi:10.1016/j.cmpb.2018.02.015

70. Yang X, Pan X, Liu H, et al. A new approach to predict lymph node metastasis in solid lung adenocarcinoma: A radiomics nomogram. *J Thorac Dis*. 2018;10(Suppl 7):S807-S819. doi:10.21037/jtd.2018.03.126

71. Zhong Y, Yuan M, Zhang T, Zhang YD, Li H, Yu TF. Radiomics approach to prediction of occult mediastinal lymph node metastasis of lung adenocarcinoma. *Am J Roentgenol*. 2018;211(1):109-113. doi:10.2214/AJR.17.19074

72. Lafata KJ, Hong JC, Geng R, et al. Association of pre-treatment radiomic features with lung cancer recurrence following stereotactic body radiation therapy. *Phys Med Biol*. 2019;64(2). doi:10.1088/1361-6560/aaf5a5

73. Akinci D’Antonoli T, Farchione A, Lenkowicz J, et al. CT Radiomics Signature of Tumor and Peritumoral Lung Parenchyma to Predict Nonsmall Cell Lung Cancer Postsurgical Recurrence Risk. *Acad Radiol*. 2020;27(4):497-507. doi:10.1016/j.acra.2019.05.019

74. He L, Huang Y, Yan L, Zheng J, Liang C, Liu Z. Radiomics-based predictive risk score: A scoring system for preoperatively predicting risk of lymph node metastasis in patients with resectable non-small cell lung cancer. *Chinese J Cancer Res*. 2019;31(4):641-652. doi:10.21147/j.issn.1000-9604.2019.04.08

75. Xu X, Huang L, Chen J, et al. Application of radiomics signature captured from pretreatment thoracic CT to predict brain metastases in stage III/IV ALK-positive non-small cell lung cancer patients. *J Thorac Dis*. 2019;11(11):4516-4528. doi:10.21037/jtd.2019.11.01

76. Ferreira-Junior JR, Koenigkam-Santos M, Magalhães Tenório AP, et al. CT-based radiomics for prediction of histologic subtype and metastatic disease in primary malignant lung neoplasms. *Int J Comput Assist Radiol Surg*. 2020;15(1):163-172. doi:10.1007/s11548-019-02093-y

77. Cong M, Feng H, Ren JL, et al. Development of a predictive radiomics model for lymph node metastases in pre-surgical CT-based stage IA non-small cell lung cancer. *Lung Cancer*. 2020;139:73-79. doi:10.1016/j.lungcan.2019.11.003

78. Coroller TP, Agrawal V, Narayan V, et al. Radiomic phenotype features predict pathological response in non-small cell lung cancer. *Radiother Oncol*. 2016;119(3):480-486. doi:10.1016/j.radonc.2016.04.004

79. Huang Y, Liu Z, He L, et al. Radiomics Signature: A Potential Biomarker for the Prediction of Disease-Free Survival in Early-Stage (I or II) Non—Small Cell Lung Cancer. *Radiology*. 2016;281(3):947-957. doi:10.1148/radiol.2016152234

80. Song J, Dong D, Huang Y, Zang Y, Liu Z, Tian J. Association between tumor heterogeneity and progression-free survival in non-small cell lung cancer patients with EGFR mutations undergoing tyrosine kinase inhibitors therapy. *Proc Annu Int Conf IEEE Eng Med Biol Soc EMBS*. 2016;2016-Octob:1268-1271. doi:10.1109/EMBC.2016.7590937

81. Coroller TP, Agrawal V, Huynh E, et al. Radiomic-Based Pathological Response Prediction from Primary Tumors and Lymph Nodes in NSCLC. *J Thorac Oncol*. 2017;12(3):467-476. doi:10.1016/j.jtho.2016.11.2226

82. Tunali I, Gray JE, Qi J, et al. Novel clinical and radiomic predictors of rapid disease progression phenotypes among lung cancer patients treated with immunotherapy: An early report. *Lung Cancer*. 2019;129:75-79. doi:10.1016/j.lungcan.2019.01.010

83. Moran A, Daly ME, Yip SSF, Yamamoto T. Radiomics-based Assessment of Radiation-induced Lung Injury After Stereotactic Body Radiotherapy. *Clin Lung Cancer*. 2017;18(6):e425-e431. doi:10.1016/j.cllc.2017.05.014

84. Krafft SP, Rao A, Stingo F, et al. The utility of quantitative CT radiomics features for improved prediction of radiation pneumonitis. *Med Phys*. 2018;45(11):5317-5324. doi:10.1002/mp.13150

85. Yuan M, Liu JY, Zhang T, Zhang YD, Li H, Yu TF. Prognostic Impact of the Findings on Thin-Section Computed Tomography in stage 1 lung adenocarcinoma with visceral pleural invasion. *Sci Rep*. 2018;8(1):4743. doi:10.1038/s41598-018-22853-1

86. Yang M, Ren Y, She Y, et al. Imaging phenotype using radiomics to predict dry pleural dissemination in non-small cell lung cancer. *Ann Transl Med*. 2019;7(12):259. doi:10.21037/atm.2019.05.20

87. Aerts HJWL, Grossmann P, Tan Y, et al. Defining a Radiomic Response Phenotype: A Pilot Study using targeted therapy in NSCLC. *Sci Rep*. 2016;6:33860. doi:10.1038/srep33860

88. Rios Velazquez E, Parmar C, Liu Y, et al. Somatic mutations drive distinct imaging phenotypes in lung cancer. *Cancer Res*. 2017;77(14):3922-3930. doi:10.1158/0008-5472.CAN-17-0122

89. Mei D, Luo Y, Wang Y, Gong J. CT texture analysis of lung adenocarcinoma: Can Radiomic features be surrogate biomarkers for EGFR mutation statuses. *Cancer Imaging*. 2018;18(1):52. doi:10.1186/s40644-018-0184-2

90. Digumarthy SR, Padole AM, Gullo R Lo, Sequist L V., Kalra MK. Can CT radiomic analysis in NSCLC predict histology and EGFR mutation status? *Medicine (Baltimore)*. 2019;98(1):e13963. doi:10.1097/MD.0000000000013963

91. Jia TY, Xiong JF, Li XY, et al. Identifying EGFR mutations in lung adenocarcinoma by noninvasive imaging using radiomics features and random forest modeling. *Eur Radiol*. 2019;29(9):4742-4750. doi:10.1007/s00330-019-06024-y

92. Li S, Ding C, Zhang H, Song J, Wu L. Radiomics for the prediction of EGFR mutation subtypes in non-small cell lung cancer. *Med Phys*. 2019;46(10):4545-4552. doi:10.1002/mp.13747

93. Tu W, Sun G, Fan L, et al. Radiomics signature: A potential and incremental predictor for EGFR mutation status in NSCLC patients, comparison with CT morphology. *Lung Cancer*. 2019;132:28-35. doi:10.1016/j.lungcan.2019.03.025

94. Yang X, Dong X, Wang J, et al. Computed Tomography-Based Radiomics Signature: A Potential Indicator of Epidermal Growth Factor Receptor Mutation in Pulmonary Adenocarcinoma Appearing as a Subsolid Nodule. *Oncologist*. 2019;24(11):e1156-e1164. doi:10.1634/theoncologist.2018-0706

95. Wang X, Kong C, Xu W, et al. Decoding tumor mutation burden and driver mutations in early stage lung adenocarcinoma using CT-based radiomics signature. *Thorac cancer*. 2019;10(10):1904-1912. doi:10.1111/1759-7714.13163

96. Bak SH, Park H, Lee HY, et al. Imaging genotyping of functional signaling pathways in lung squamous cell carcinoma using a radiomics approach. *Sci Rep*. 2018;8(1):1-9. doi:10.1038/s41598-018-21706-1

97. Patil R, Mahadevaiah G, Dekker A. An Approach Toward Automatic Classification of Tumor Histopathology of Non–Small Cell Lung Cancer Based on Radiomic Features. *Tomography*. 2016;2(4):374-377. doi:10.18383/j.tom.2016.00244

98. Wu W, Parmar C, Grossmann P, et al. Exploratory Study to Identify Radiomics Classifiers for Lung Cancer Histology. *Front Oncol*. 2016;6(71):1-11. doi:10.3389/fonc.2016.00071

99. Zhu X, Dong D, Chen Z, et al. Radiomic signature as a diagnostic factor for histologic subtype classification of non-small cell lung cancer. *Eur Radiol*. 2018;28(7):2772-2778. doi:10.1007/s00330-017-5221-1

100. E L, Lu L, Li L, Yang H, Schwartz LH, Zhao B. Radiomics for Classifying Histological Subtypes of Lung Cancer Based on Multiphasic Contrast-Enhanced Computed Tomography. *J Comput Assist Tomogr*. 2019;43(2):300-306. doi:10.1097/RCT.0000000000000836

101. Liu J, Cui J, Liu F, Yuan Y, Guo F, Zhang G. Multi-subtype classification model for non-small cell lung cancer based on radiomics: SLS model. *Med Phys*. 2019;46(7):3091-3100. doi:10.1002/mp.13551

102. Zhou B, Xu J, Tian Y, Yuan S, Li X. Correlation between radiomic features based on contrast-enhanced computed tomography images and Ki-67 proliferation index in lung cancer: A preliminary study. *Thorac Cancer*. 2018;9(10):1235-1240. doi:10.1111/1759-7714.12821

103. Gu Q, Feng Z, Liang Q, et al. Machine learning-based radiomics strategy for prediction of cell proliferation in non-small cell lung cancer. *Eur J Radiol*. 2019;118:32-37. doi:10.1016/j.ejrad.2019.06.025

104. Song SH, Park H, Lee G, et al. Imaging Phenotyping Using Radiomics to Predict Micropapillary Pattern within Lung Adenocarcinoma. *J Thorac Oncol*. 2017;12(4):624-632. doi:10.1016/j.jtho.2016.11.2230

105. Chen X, Fang M, Dong D, et al. A Radiomics Signature in Preoperative Predicting Degree of Tumor Differentiation in Patients with Non–small Cell Lung Cancer. *Acad Radiol*. 2018;25(12):1548-1555. doi:10.1016/j.acra.2018.02.019

106. She Y, Zhang L, Zhu H, et al. The predictive value of CT-based radiomics in differentiating indolent from invasive lung adenocarcinoma in patients with pulmonary nodules. *Eur Radiol*. 2018;28(12):5121-5128. doi:10.1007/s00330-018-5509-9

107. Yang B, Guo L, Lu G, Shan W, Duan L, Duan S. Radiomic signature: a non-invasive biomarker for discriminating invasive and non-invasive cases of lung adenocarcinoma. *Cancer Manag Res*. 2019;11:7825-7834. doi:10.2147/CMAR.S217887

108. Collins GS, Reitsma JB, Altman DG, Moons KGM. Transparent reporting of a multivariable prediction model for individual prognosis or diagnosis (TRIPOD): The TRIPOD Statement. *Eur Urol*. 2015;67(6):1142-1151. doi:10.1016/j.eururo.2014.11.025

109. Lambin P, Leijenaar RTH, Deist TM, et al. Radiomics: The bridge between medical imaging and personalized medicine. *Nat Rev Clin Oncol*. 2017;14(12):749-762. doi:10.1038/nrclinonc.2017.141
